# Protein Kinase C δ Regulates the Depletion of Actin at the Immunological Synapse Required for Polarized Exosome Secretion by T Cells

**DOI:** 10.3389/fimmu.2019.00851

**Published:** 2019-04-26

**Authors:** Gonzalo Herranz, Pablo Aguilera, Sergio Dávila, Alicia Sánchez, Bianca Stancu, Jesús Gómez, David Fernández-Moreno, Raúl de Martín, Mario Quintanilla, Teresa Fernández, Pablo Rodríguez-Silvestre, Laura Márquez-Expósito, Ana Bello-Gamboa, Alberto Fraile-Ramos, Víctor Calvo, Manuel Izquierdo

**Affiliations:** ^1^Departamento de Bioquímica, Instituto de Investigaciones Biomédicas Alberto Sols CSIC-UAM, Madrid, Spain; ^2^Departamento de Biología Celular, Facultad de Medicina, Universidad Complutense de Madrid, Madrid, Spain

**Keywords:** T lymphocytes, immune synapse, protein kinase C δ, multivesicular bodies, exosomes, cytotoxic activity, cell death

## Abstract

Multivesicular bodies (MVB) are endocytic compartments that enclose intraluminal vesicles (ILVs) formed by inward budding from the limiting membrane of endosomes. In T lymphocytes, ILVs are secreted as Fas ligand-bearing, pro-apoptotic exosomes following T cell receptor (TCR)-induced fusion of MVB with the plasma membrane at the immune synapse (IS). In this study we show that protein kinase C δ (PKCδ), a novel PKC isotype activated by diacylglycerol (DAG), regulates TCR-controlled MVB polarization toward the IS and exosome secretion. Concomitantly, we demonstrate that PKCδ-interfered T lymphocytes are defective in activation-induced cell death. Using a DAG sensor based on the C1 DAG-binding domain of PKCδ and a GFP-PKCδ chimera, we reveal that T lymphocyte activation enhances DAG levels at the MVB endomembranes which mediates the association of PKCδ to MVB. Spatiotemporal reorganization of F-actin at the IS is inhibited in PKCδ-interfered T lymphocytes. Therefore, we propose PKCδ as a DAG effector that regulates the actin reorganization necessary for MVB traffic and exosome secretion.

## Introduction

T cell receptor (TCR) stimulation by antigen presented by major histocompatibility complex (MHC) molecules on an antigen-presenting cell (APC) induces the formation of the immunological synapse (IS), the convergence of the secretory granules of T lymphocytes toward the microtubule-organizing center (MTOC) and, almost simultaneously, the polarization of the MTOC to the IS (1, 2). This ensures the specificity of T cell effector responses by enabling polarized secretory traffic toward the APC (1, 2), spatially and temporally focusing secretion at the synaptic cleft (3). The polarization of the MTOC toward the IS is conducted by a transient increase in cortical actin at the IS, followed by a decrease in cortical actin density at the central region of the immune synapse (cIS) that contains the secretory domain. The central supramolecular activation cluster (cSMAC) next to this secretory domain is also located within the F-actin-low region at cIS (Fact-low cIS) (2, 4, 5). In parallel, F-actin accumulation occurs at the edge of the T lymphocyte/APC interface, which constitutes the distal SMAC (dSMAC) and delimits the synaptic contact region (6, 7). The secretory granules from cytotoxic T lymphocytes (CTL) (also called “lytic” or “cytotoxic” granules) contain diverse apoptosis-inducing molecules (8), including Fas ligand (FasL). Among several pro-apoptotic mechanisms, CTL kill Fas^+^ target cells by rapidly exposing intact, pre-formed FasL on the plasma membrane at the IS (9). FasL induces cross-linking of the Fas death receptor on the target cell and subsequent apoptosis (10). In resting CTL, FasL is located at the limiting membrane of secretory multivesicular bodies (MVB) (9). In addition, FasL can be sorted from the limiting membrane of the MVB to the intraluminal vesicles (ILV) via inward budding, which occurs during maturation of MVB in CTL, CD4+ T lymphoblasts and Jurkat, a CD4+ T helper (Th) type cell line (11–13). Upon TCR activation of CTL and MTOC reorientation, lytic granules undergo fusion with the plasma membrane at the IS (5). As a consequence, two mechanisms for the transport of pro-apoptotic FasL to the extracellular milieu may coexist: relocalization of FasL to the cell surface (9), and secretion of FasL-containing ILV as lethal extracellular nanovesicles (50–100 nm size) called exosomes (13–15). While exosomes are constitutively secreted by a variety of cell lineages and tumor cells, in T and B lymphocytes exosome secretion is triggered upon activation of cell surface receptors, which in turn regulates antigen-specific immune responses (16). Exosomes are involved in important processes related to TCR-triggered immune responses, including T lymphocyte-mediated cytotoxicity, activation-induced cell death (AICD) of CD4+ lymphocytes, antigen presentation, intercellular miRNA exchange (11–13, 17, 18) and thymic development (19). However, the mechanisms underlying MVB traffic and exosome secretion are poorly understood. In this context, it is known that MTOC reorientation in CTL is initially guided by a diacylglycerol (DAG) gradient centered at the IS (20), which is *de novo* produced by TCR-stimulated phospholipase C (PLC) activation. DAG activates, among others, several members of the protein kinase C (PKC) and the protein kinase D (PKD) families (21). Phosphorylation of DAG by diacylglycerol kinase α (DGKα) to produce phosphatidic acid (PA) (22) is one of the mechanisms involved in the spatiotemporal control of the DAG gradient (23) and MTOC reorientation to the IS (20). Furthermore, several authors have described DGKα as a crucial factor in the polarization of late endosomes/MVB (24). We have shown that DGKα controls the polarized secretion of exosomes containing FasL in Th lymphocytes (13, 25) and that the kinase activity of DGKα inhibits ILV formation during MVB maturation (25). In addition, we have identified a DAG-activated enzyme, PKD1/2, as a key component of this DGKα-controlled pathway involved in MVB maturation and exosome secretion (26). Besides this early regulation, DGKα also controls MTOC and MVB polarization toward the IS both in CTL and CD4^+^ T lymphocytes (20, 25, 27), although the molecular basis underlying this second checkpoint remains unclear. The fact that the novel PKC family member PKCδ, a DAG-activated PKC isotype, is necessary for the polarization of lytic granules and cytotoxicity in mouse CTL (28, 29) prompted us to study the function of PKCδ in MVB polarized trafficking and exosome secretion in human T lymphocytes.

## Materials and Methods

### Cells

J-HM1-2.2 Jurkat cells expressing human muscarinic type 1 receptor (HM1R) and high levels of PKCδ have been used as a model system to trigger phosphatidylinositol turnover and DAG production at the plasma membrane upon carbachol (CCH) stimulation (30). Raji B and Jurkat T (clone JE6.1) cell lines were obtained from the ATCC. Cell lines were cultured in RPMI 1640 medium containing L-glutamine (Invitrogen) with 10% heat-inactivated FCS (Gibco) and penicillin/streptomycin (Gibco). Jurkat cells (clone JE6.1) transfected with control and PKCδ shRNA-encoding plasmids were selected with puromycin (1 μg/ml) and clones isolated by limiting dilution. Human primary T lymphoblasts from healthy volunteers were obtained and cultured as described previously (31).

### ShRNA Plasmids, Expression Vectors, Transfection Assays, and Isolation of Clones

Plasmids used in this study were as follows: pEFbos-GFP was described previously (13, 23); pEFGFP-C1bosCD63 and pECFP-C1CD63 were provided by G. Griffiths; mouse pEGFP-PKCδwt (GFP-PKCδWT), pEGFP-PKCδDR144/145A constitutively active mutant (GFP-PKCδCA) (32) and pEGFP-PKCδK376A kinase-dead mutant (GFP-PKCδKD) (33, 34) were obtained from A. Zweifach and D. M. Reyland. GFP-C1bPKCθ expression plasmid was kindly provided by I. Mérida; UpwardDAG2 (U.DAG2) (35) was generously provided by A.M. Quinn (Montana Molecular Inc.). In some experiments, human DGKα was silenced using the pSUPER RNAi System (pSR-GFP bicistronic or pSuperplasmids; Oligoengine, Seattle, WA, USA) with the appropriate hairpin as described (25). pDsRed2-PKD1wt plasmid was previously described (26). U.DAG2 is a genetically encoded, fluorescent protein-containing DAG sensor based on the insertion of the circularly permuted (cp) EGFP into a PKCδ coding sequence that was modified by deleting only the N-terminal region containing the C2 domain (35). The U.DAG2 sensor maintains the C1, DAG-binding domain and the catalytic domain of PKCδ and, upon DAG production, is recruited to cellular membranes following DAG binding and undergoes conformational changes, leading to a rapid fluorescence increase (35, 36). This sensor was demonstrated to produce rapid, robust and reversible changes in green fluorescence in a live-cell assay (35).

Control short-hairpin RNA (Cont shRNA) plasmid-A (Santa Cruz Biotechnology), PKCδ shRNA plasmid (h) (Santa Cruz Biotechnology) or a mixture of three pSIREN-RetroQ retroviral vectors (Clontech) encoding shRNAs against human PKCδ (37) were used to generate stable JE6.1 Jurkat clones. All these plasmids expressed a puromycin resistance gene for the selection of stably transfected clones. The plasmids were verified by sequencing. For characterization of control and PKCδ-interfered Jurkat stable clones, PKCδ levels were analyzed by WB and cell surface levels of CD3/TCR, CD2, CD4, LFA-1, CD28, CD45, and CD95 (Fas) were analyzed by flow cytometry after expansion of the cell clones obtained by limiting dilution. For transient transfection experiments, J-HM1-2.2 and Jurkat clones were transiently transfected with 20–30 μg of the plasmids as described (13). For exosome secretion experiments, mouse PKCδ expression constructs were transiently co-transfected with exosome reporter GFP-CD63 expression plasmid in a 3:1 molecular ratio (26). Human primary T lymphoblasts were cultured in the presence of IL-2 as previously described (31) and were transfected, between 3 and 7 days after the addition of IL-2, with 2 μg of the indicated expression and interference plasmids, by using an appropriate nucleofector kit (Amaxa® Human T Cell Nucleofector® Kit, Program T-20 or T-23 for Nucleofector® I).

### Antibodies and Reagents

Antibodies used in this study were obtained from the indicated sources: rabbit monoclonal anti-human PKCδ EP1486Y (Abcam) for WB (this antibody does not recognize mouse PKCδ); rabbit polyclonal anti-rat PKCδ C-17 (Santa Cruz Biotechnology) for WB (recognizes both human and mouse PKCδ); anti-human CD3 UCHT1 (BD Biosciences and Santa Cruz Biotechnology) for cell stimulation and immunofluorescence; rabbit polyclonal anti-phospho-PKCδThr505 (Cell Signaling Technology) for WB; mouse monoclonal anti-CD63 clone NKI-C-3 (Oncogene) for WB; mouse monoclonal anti-CD63 clone TA3/18 (Immunostep) for immunofluorescence; and mouse monoclonal anti-γ-tubulin (SIGMA) for immunofluorescence. Fluorochrome-coupled secondary antibodies (goat-anti-mouse IgG AF488 A-11029, goat-anti-rabbit IgG AF488 A-11034, goat-anti-mouse IgG AF546 A-11030, goat-anti-mouse IgG AF647 A-21236) for immunofluorescence were from ThermoFisher. All horseradish peroxidase (HRP)-coupled secondary Abs (goat anti-mouse IgG-HRP, sc-2005 and goat anti-rabbit IgG-HRP, sc-2004) were obtained from Santa Cruz Biotechnology. Cell tracker blue (CMAC) and phalloidin were from ThermoFisher. Annexin V-PE was from Immunostep. Carbachol (CCH) and staphylococcal enterotoxin E (SEE) were from SIGMA and Toxin Technology, Inc (USA), respectively. Blocking antibody directed against CD95 (Fas), clone DX2, was from BDBiosciences.

### Isolation and Quantitation of Exosomes

Exosomes produced by equal numbers of cells for each experimental condition were isolated from cell culture supernatants as previously described (14, 15, 26). No significant differences in β-actin levels (i.e., **Figure 3**) were observed in the lysates of cells, stimulated or not, at the end of the cell culture period for exosome secretion, showing that the exosomes were produced by equal numbers of viable cells. Using these standard protocols, culture supernatants of 20 x 10^6^ Jurkat cells were centrifuged in sequential steps to eliminate cells and cell debris/apoptotic bodies (38), and the exosomes were recovered by ultracentrifugation (100,000xg for 12 h) as described (14). In some experiments, to quantify exosomes and to analyze their size distribution, the cell culture supernatant collected just before the ultracentrifugation step was diluted (1/5) in Hank's balanced salt solution (HBSS) and analyzed by Nanoparticle Tracking Analysis (NTA) with the use of NANOSIGHT equipment (LM10, Malvern) that was calibrated with 50 nm, 100 nm and 400 nm fluorescent calibration beads (Malvern). The hydrodynamic diameter measured by NTA, although apparently higher than that originally described for exosomes using electron microscopy (50–100 nm), certainly corresponds to the real size of canonic, unfixed exosomes in solution, as described (39). The NTA measurements of exosome concentration (particles/ml) were normalized by the exosome-producing cell number, by referring exosome concentration to β-actin or endogenous CD63 signals in the WB of the cell lysates. CD63 is characteristically present in MVB, ILVs and hence in exosomes, but also in secretory lysosomes and the plasma membrane. Plasma membrane CD63 localization is produced by degranulation of MVB and diffusion of CD63 from the limiting membrane of MVB to the plasma membrane upon MVB fusion (25, 26). This protein and its chimeras (GFP-CD63) have been used as appropriate reporters for MVB/exosomes (13, 40, 41), and allow the quantitation of exosome secretion in Jurkat cells and primary human T lymphoblasts (13, 25, 38). To analyze the exosomes from cells expressing the exosome reporter GFP-CD63, a similar protocol was performed, although 1 × 10^6^ Jurkat cells or human T lymphoblasts were used and the WB signals in exosome lysates were normalized by the expression levels of GFP-CD63 among different transfections and stimuli in the WB corresponding to the cell lysates (25, 41). It has been established that the exosomal CD63 WB signal correlates well with the results of exosome number obtained by flow cytometry (42), by electron microscopy (43) and by nanoparticle concentration analysis (nanoparticles/ml), using NTA (26). Thus, WB analysis of endogenous or GFP-tagged CD63 in isolated exosomes constitutes a *bona fide* method to measure exosome production (25, 26).

### Western Blot Analysis of Cell and Exosome Lysates

Cells and isolated exosomes were lysed in RIPA lysis buffer containing protease inhibitors. Approximately 50 μg of exosomal proteins was recovered in the 100,000xg pellet from 20 × 10^6^ cells. Exosomes were resuspended in 60 μl of RIPA lysis buffer and 20 μl of exosomal or cell lysates were separated on SDS-PAGE under reducing conditions and transferred to Hybond™ ECL™ membranes (GE Healthcare). For CD63 detection, proteins were separated under non-reducing conditions as described (13). For WB analysis of exosomes, each lane contained the total exosomal protein that was recovered in the culture medium from the same number of cells, untreated or treated with stimuli. Blots were incubated with mouse anti-CD63 (clone NKI-C-3, Oncogene) and developed with the appropriate HRP-conjugated secondary antibody using enhanced chemiluminescence (ECL). Autoradiography films were scanned and the bands were quantified with the use of Quantity One 4.4.0 (Bio-Rad) and ImageJ (Rasband, W.S., ImageJ, National Institutes of Health, Bethesda, Maryland, USA, http://rsb.info.nih.gov/ij/, 1997-2004) software.

### Time-Lapse Microscopy, Immunofluorescence Experiments, and Image Analysis

**Figure 1:**
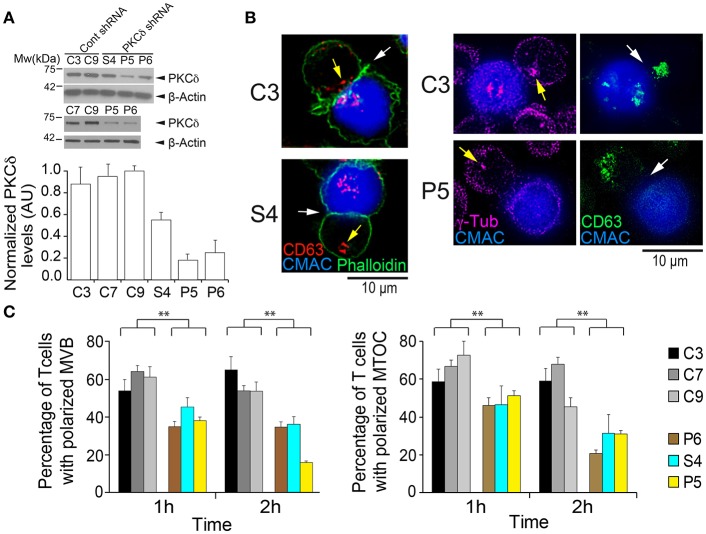
PKCδ regulates the polarization of the MTOC and MVB. **(A)** PKCδ expression levels in different control (C3, C7, and C9) and PKCδ-interfered (S4, P5, and P6) Jurkat clones. Cell lysates were analyzed by WB with anti-PKCδ antibody, and β-actin was used as a loading control. In the lower panel, normalized levels of PKCδ expressed as arbitrary units (AU), means plus standard deviations (SD) (*n* = 3), is represented. **(B)** C3, C7, C9 (control) and S4, P5, P6 (PKCδ-interfered) Jurkat clones were challenged with CMAC-labeled SEE-pulsed Raji cells to induce synaptic conjugate formation. After 1 h cells were fixed, permeabilized, stained with phalloidin and anti-CD63 or anti-γ-tubulin Abs, and imaged by fluorescence microscopy to analyze MVB and MTOC polarization toward the IS. Representative examples of the polarized C3 control clone and non-polarized (S4 and P5) PKCδ-interfered clones are shown. White arrows indicate the IS areas and yellow arrows CD63^+^ MVB or MTOC. CMAC labeling of Raji cells in blue, phalloidin in green, CD63 in red in left panels and green in right panels and anti-γ-tubulin in magenta. Scale bars, 10 μm.) **(C)** The percentage of the control and PKCδ-interfered Jurkat clones forming synapses with polarized MVB (left) and MTOC (right) after 1 and 2 h challenge with SEE-pulsed Raji cells was determined as indicated in the Materials and Methods section by measuring the respective MVB and MTOC polarization indexes in control and PKCδ-interfered Jurkat clones (see also Supplementary Figure 1). Data are means plus SD (*n* = 3, analyzing at least 50 synapses from 15 different microscopy fields per experiment). Single-factor analysis of variance (ANOVA) was performed between the indicated groups. ^**^p ≤ 0.05.

**Figure 2:**
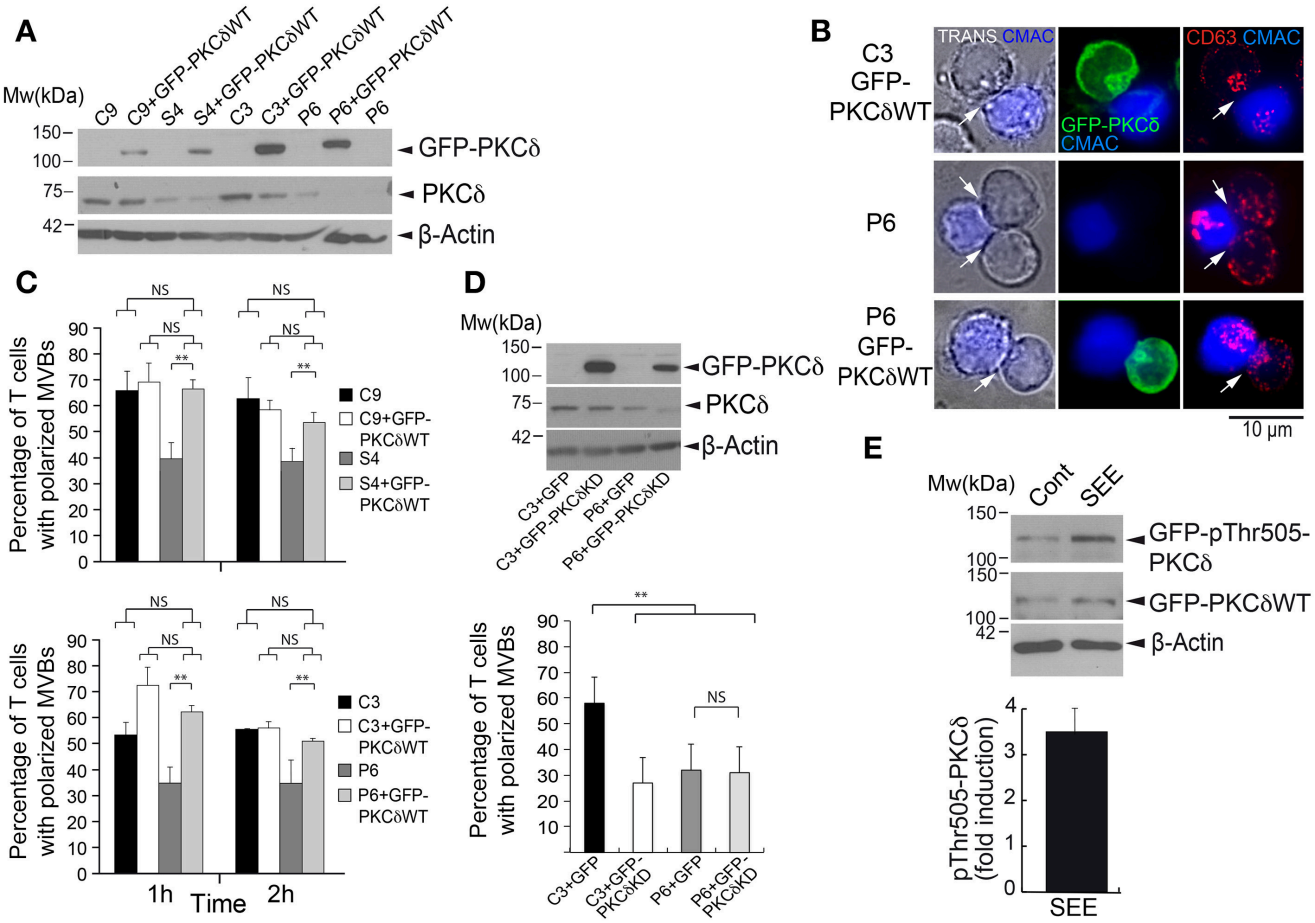
GFP-PKCδ expression restores the polarization of MVB. C3 and C9 control and S4 and P6 PKCδ-interfered Jurkat clones were transfected or not with GFP, GFP-PKCδWT, or GFP-PKCδKD mutant. **(A)** WB of lysates from C3, C9, S4, and P6 clones, transfected or not with GFP-PKCδWT, was carried out with an anti-ratPKCδ, anti-humanPCKδ and anti-β-actin Abs. **(B)** Cells were challenged with CMAC-labeled SEE-pulsed Raji cells to induce synaptic conjugate formation. After 1 h cells were fixed, permeabilized, stained with anti-CD63 Abs and imaged by fluorescence microscopy. Transmittance (TRANS) plus CMAC is shown in the left panel, and the white arrows indicate the IS areas. CMAC labeling of Raji cells in blue, GFP-PKCδ in green and CD63 in red. Representative examples of polarized C3 GFP-PKCδWT^+^ clone, non-polarized P6 clone and polarized P6 GFP-PKCδWT^+^ clone are shown. Scale bar, 10 μm. **(C)** Quantification of MVB polarization in C9, C3 (control) and S4, P6 (PKCδ-interfered) Jurkat clones transfected or not with GFP-PKCδWT and challenged with CMAC-labeled, SEE-pulsed Raji cells for 1 and 2 h. The percentage of synapse-forming clones with polarized MVB, expressing or not expressing GFP-PKCδWT, was determined as indicated in Materials and Methods section (see also Supplementary Figure 1). Data are means plus SD (*n* = 3, analyzing at least 50 synapses from 15 different microscopy fields per experiment). Single-factor ANOVA was performed between the indicated groups. NS, not significant, ^**^p ≤ 0.05. **(D)** Upper panel: Lysates of C3 and P6 clones transfected with either GFP or GFP-PKCδKD mutant were analyzed by WB with an anti-ratPKCδ, anti-humanPKCδ and anti-β-actin Abs. Lower panel: quantification of MVB polarization efficiency in C3 control and P6 PKCδ-interfered Jurkat clones transfected with either GFP or GFP-PKCδKD and challenged with CMAC-labeled, SEE-pulsed Raji cells for 1 h. The percentage of synapse-forming clones with polarized MVB, expressing or not expressing GFP-PKCδKD, was determined as in **(C)** Data are means plus SD (*n* = 3, analyzing at least 40 synapses from 15 different microscopy fields per experiment) and single-factor ANOVA was performed between the indicated groups. NS, not significant, ^**^p ≤ 0.05. **(E)** C3 control Jurkat clone was transfected with GFP-PKCδWT and challenged with non-pulsed (Cont) or SEE-pulsed (SEE) Raji cells for 1 h. Cells were lysed and lysates analyzed by WB with anti-phosphoThr505-PKCδ, anti-rat PKCδ Ab (C-17) and anti-β-actin to normalize. In the lower panel, mean fold induction plus SD (*n* = 3) of normalized phosphoThr505-PKCδ signal is represented.

Jurkat clones transfected with the different expression plasmids were attached to glass-bottom IBIDI microwell culture dishes using fibronectin (0.1 mg/ml) at 24–48 h post-transfection and stimulated in culture medium at 37°C. In some experiments, Raji cells attached to glass-bottom IBIDI microwell culture dishes using fibronectin were labeled with CMAC and pulsed with 1 μg/ml SEE, and then mixed with transfected Jurkat clones and the immune synapses were analyzed as described (25). In other experiments, transfected Jurkat clones were stimulated with plastic-bound anti-TCR UCHT1 Ab (10 μg/ml) or directly in suspension with CCH (500 μM) or phorbol myristate acetate **(**PMA, 100 ng/ml). Immunofluorescence of fixed synapses was performed as previously described (44), and additional fixation was made between each fluorochrome-coupled secondary antibody and subsequent fluorochrome-coupled primary antibody staining, to exclude any potential cross-reaction of secondary antibodies. Time-lapse experiments were performed using an OKO-lab stage incubator (OKO) on a Nikon Eclipse TiE microscope equipped with a DS-Qi1MC digital camera and a PlanApo VC 60x NA 1.4 objective (NIKON). Time-lapse acquisition and analysis were performed by using NIS-AR software (NIKON). Subsequently, epi-fluorescence images were improved by Huygens Deconvolution Software from Scientific Volume Image (SVI) using the “widefield” optical option. Deconvolution is a computational image processing technique that can improve image resolution and contrast up to two times, down to 150–100 nm in XY and 500 nm in Z-axis (https://svi.nl/Deconvolution). Deconvolution requires the knowledge of the idealized or measured point spread function (PSF) of the microscope and the imaging technique used (45). One example of the power of deconvolution applied to epifluorescence videos on the polarized traffic of MVB at the IS is provided (25), (video before deconvolution, https://www.youtube.com/watch?v=mID0m3usQOQ; video after deconvolution, https://www.youtube.com/watch?v=Aj0vPj6WAII). For quantification, digital images were analyzed using NIS-AR (Nikon) or ImageJ software (Rasband, W.S., ImageJ, National Institutes of Health, Bethesda, Maryland, USA, http://rsb.info.nih.gov/ij/, 1997-2004). For quantification of relative fluorescence intensity (FI) in time-lapse experiments, analysis of average FI in floating regions of interest (ROI) (i.e., ROI changing over time) was performed using NIS-AR software. These measurements were performed in deconvoluted time-lapse series because of the enhanced signal-to-noise ratio of the images, although raw time-lapse series yielded comparable results. In several experiments, to define the central immune synapse region (cIS), subcellular relocalization of DsRed2-PKD1 to the synapse was evaluated in parallel (i.e., Supplementary Video 6), since PKD1 relocalizes to central synapse and cSMAC via DAG binding (46). Confocal microscopy imaging was performed by using a SP8 Leica confocal microscope, with sequential acquisition, bidirectional scanning and the following laser lines: UV (405 nm, intensity: 33.4%), supercontinuum visible (633 nm, intensity: 15.2%), supercontinuum visible (550 nm, intensity: 20.8%), supercontinuum visible (488 nm, intensity: 31.2%). Deconvolution of confocal images was performed by using Huygens Deconvolution Software from Scientific Volume Image (SVI) with the “confocal” optical option. Colocalization analyses were accomplished by using the Jacop plugin from ImageJ. The velocity of movement of MVB was measured by analyzing the trajectories of CFP-CD63^+^ vesicles in videos with the use of NIS-AR software and the ImageJ MJTrack plugin. In polarization experiments, to establish the relative ability of the MTOC and MVB to polarize toward the IS, MTOC and MVB polarization indexes (Pol. Indexes) were calculated by measuring the distance of the cell's center of mass (cell^C^) to the IS (“B” distance), and the distances between the projections of the MTOC or MVB centers of mass (MTOC^C^ and MVB^C^, respectively) to the cell^C^ (“A” distance) (Supplementary Figure 1C). Cell^C^ position was taken as the origin to measure distances, and those “A” values in the opposite direction to the synapse were taken as negative. Pol. Indexes were calculated, as described in Supplementary Figure 1C, as the ratio of distances A and B (Pol. Index = A/B), ranging from +1 to −1. Therefore, Pol. Index values were normalized by cell size and shape (Supplementary Figure 1C). The cut-off value for Pol. Indexes was arbitrarily set up at 0.25 (synapses displaying Pol. Index >0.25 were scored as “polarized” and those with Pol. Index <0.25 as “not polarized”) (Supplementary Figure 1D). The percentage of synapses with polarized MVB/MTOC that measures polarization efficiency was calculated considering this cut-off value (Supplementary Figure 1D, Figures 1C, 2C).

The relative area of the F-actin-low region at the cIS (Fact-low cIS) was measured on confocal microscopy images using the 3D Viewer ImageJ plugin. Briefly, a 2D face on view of the synapse observing from the T lymphocyte (IS interface) (i.e., Supplementary Video 10 and **Figure 9A**) was generated, and the boundary of the T lymphocyte/APC synaptic contact is defined by the distal SMAC (dSMAC), which consists of a circular array with F-actin accumulating at the edge of the T cell/APC interface (6, 47). Thus, the IS area was delimited by the edge of the F-actin signal. When necessary, the definition of the regions of interest (ROI) to measure the areas of the F-actin-low region at the center of the IS (Fact-low cIS area) and the F-actin accumulation region at the IS (IS area) was performed by using the automated algorithm “auto-detect ROI/segmentation” from NIS-AR or by ImageJ segmentation software. Next, these defined ROI areas (Fact-low cIS area and IS area) were measured, and the relative area of the F-actin depleted region at cIS (Fact-low cIS area/IS area) was determined (Supplementary Figures 7B,C). This allowed normalization by cell size and IS contact area. The cut-off value for synapses substantiating a F-actin depleted region at cIS was arbitrarily set up at 0.1 (those synapses displaying area ratio >0.1 were scored as “depleted”; Supplementary Figure 7C). The percentage of synapses with F-actin depleted at cIS was calculated considering this cut-off value (Supplementary Figure 7C, **Figure 9C**). The plot profile analyses of FI corresponding to phalloidin along the indicated ROIs at the IS interfaces were performed by using ImageJ. Image analysis data correspond to at least three different experiments, analyzing a minimum of 30 synapses from 15 different, randomly selected microscopy fields per experiment. ANOVA analysis was performed for statistical significance of the results using Excel and IBM's SPSS Statistics software.

### Apoptosis Experiments

GFP-PKCδ transfected and untransfected Jurkat clones were challenged with CMAC-labeled, SEE-pulsed Raji cells or Raji cells as a control. After the indicated culture periods, the percentage of apoptotic cells (Annexin-V^+^ cells) was analyzed by end point, flow cytometry analysis. Since both size and complexity of Jurkat clones and Raji cells are similar it is not possible, using Forward Scatter (FS) and Side Scatter (SS), to gate cell types using these parameters. Therefore, we have only used CMAC fluorescence gating to exclude the death of Raji cells of the analyses. In some experiments, for the optimal correlation between the formation of synaptic conjugates with apoptosis measurements, these co-cultured cells were continuously imaged by time-lapse microscopy to visualize some early signs of AICD (i.e., plasma membrane blebbing and subsequent cell shrinkage) in the Jurkat clones (CMAC negative) forming stable synapses (Supplementary Video 3). In other experiments, Jurkat clones were pre-incubated for 30 min with a blocking antibody directed against human Fas (clone DX2, 1 μg/ml), before the challenge with the SEE-pulsed Raji cells.

## Results

### PKCδ Regulates the Polarized Traffic of MTOC and MVB Toward the IS

To study the role of PKCδ in the polarized traffic of MVB in human T lymphocytes, we generated several PKCδ-interfered clones. PKCδ-interference was analyzed by WB, and 3 Jurkat clones expressing PKCδ-shRNA-encoding plasmids showed a reduction in PKCδ levels (P5 and P6, and in lower extension S4, Figure 1A), when compared to Jurkat clones transfected with control shRNA plasmid (C3, C7, and C9), and were used in further studies. The PKCδ-interfered clones P5, P6, and S4 had similar levels of the cell surface molecules relevant for T lymphocyte interaction with APC when compared to control clones (Supplementary Figure 1A). We then analyzed the formation of synaptic conjugates in a human IS model with the use of CMAC-labeled Raji cells presenting superantigen (SEE). The formation of synaptic conjugates was not affected by PKCδ interference (i.e., Supplementary Video 1), and the percentage of cells undergoing conjugate formation after 1 h of challenge with SEE-pulsed APC was similar in the different clones (i.e., 67 ± 4% of C3 control clone cells underwent conjugate formation vs. 70 ± 7% of P5 PKCδ-interfered clone cells, mean±SD in 3 different experiments, not significant, Supplementary Figure 1B). Next, to analyze the traffic of MVB and to study synaptic architecture, we used an approach based on time-lapse studies combined with end point immunofluorescence analysis in fixed cells. This double approach allowed us, on one hand, to study the traffic of CD63^+^ MVB shortly after IS formation in living cells and, on the other hand, to label endogenous MVB and MTOC with anti-CD63 or anti-γ-tubulin, respectively, in fixed cells to perform end point 3D analysis of F-actin and synaptic architecture. Both approaches were complementarily required, since when the formation of synaptic conjugates was analyzed at the single cell level by time-lapse microscopy, we observed that IS formation constitutes an asynchronous (**Figure 8A**), stochastic process (26, 45), as previously shown (48). We have studied the kinetics of conjugate formation by time-lapse microscopy in control and PKCδ-interfered clones, and we observed that after 30 min of challenge only 30% of cells formed conjugates, whereas after 1 h of challenge the efficiency rose to 65% and after 2 h reached 75% (Supplementary Figure 1B). In addition to this fact, and since preliminary results with the different clones showed both the maximal conjugate formation (measured as the percentage of Jurkat cells forming synaptic conjugates) and the maximal polarization (the percentage of Jurkat cells forming synapses with polarized MVB/MTOC, see below) occurred between 1 and 2 h after challenge with APCs, we decided to use these time points in our end point experiments with fixed synapses (Figure 1). However, it should be pointed out that these time points indicate only the period elapsed after Jurkat clone addition to the SEE-pulsed APCs, but not the beginning of the conjugate formation period (**Figure 8A**). For all these reasons, end point analysis in fixed cells provided neither temporal information regarding the onset of these synapses nor a dynamic, realistic view of the progression of MVB convergence and MVB/MTOC polarization (48), however this was indeed achieved by time-lapse studies (45) (i.e., Supplementary Video 1). For both approaches, CD63 staining was analyzed since this molecule is the canonical marker of MVB and exosomes across a multitude of cell types (http://exocarta.org/exosome_markers_new). Thus, we analyzed the polarization of MVB in a human IS model with the use of CMAC-labeled Raji cells presenting superantigen (SEE) to challenge the different Jurkat clones, either untransfected or expressing the MVB reporter CFP-CD63 (25). Time-lapse microscopy showed that upon IS formation, CFP-CD63 vesicles in the C3 control clone progressively accumulate in the vicinity of the IS, as previously reported (25, 26). This feature was not observed in the P5 PKCδ-interfered clone (Supplementary Video 1, Supplementary Figure 2A). End point analysis of the polarization of endogenous CD63^+^ vesicles and the MTOC were performed in synapses established by the different clones during the two different times (1 and 2 h) after challenge with SEE-presenting Raji cells (Figures 1B,C). In Figure 1B, images of polarized (C3) and non-polarized (S4 and P5) clones forming synapses are shown, representing the data obtained when all control (C3, C7 and C9) and PKCδ-interfered clones (P6, P5, and S4) were compared (Figure 1C, Supplementary Figure 1D). The MVB and MTOC Pol. Indexes were determined and the percentage of synapses with polarized MVB/MTOC (i.e. synapses substantiating Pol. Indexes >0.25) was calculated (Supplementary Figure 1). Despite remarkable dispersion in Pol. Index values for each clone, significant differences among all the control and all the PKCδ-interfered clones existed (Supplementary Figures 1C,D). PKCδ-interfered clones forming IS exhibited lower polarization percentages (15–50%) for both MVB and the MTOC when compared with control clones (50–75%) (Figure 1C). Remarkably, we found a strong lineal correlation between MVB and MTOC Pol. Indexes for each clone (i.e. Pearson's lineal correlation coefficient of 0.962 and 0.949 for C3 and P5 clones, respectively). Furthermore, in all the analyzed synapses the MTOC^C^ was coincident or very proximal to the MVB^C^ (i.e., white crosses in Supplementary Figure 1C), regardless of polarization. In addition, when we continuously analyzed the Pol. Index during 4 and 15 h of synapse formation in time-lapse experiments, the lower efficiency in MVB polarization in PKCδ-interfered clones was maintained (Supplementary Figure 2B). No significant differences were found between the average velocity of MVB movement in the PKCδ-interfered clones when compared with that of control clones (mean ± SD = 3.2 ± 0.4 μm/s and 3.1 μm/s ±0.3 for C3 and P5 clones, respectively, analyzing 200 CFP-CD63^+^ vesicles per clone, not significant). Therefore, PKCδ-interfered clones exhibited a continuously reduced ability to polarize the secretory machinery toward the IS, although the velocity of MVB remained unaffected. To determine the specificity of PKCδ silencing in MVB polarization, we carried out rescue experiments with a mouse PKCδ GFP-tagged construct resistant to shRNA inhibition (Figure 2A). Untransfected or GFP-PKCδ-transfected control and PKCδ-interfered Jurkat clones were challenged with SEE-pulsed Raji cells to induce IS formation, and the MVB polarization was analyzed by time-lapse microscopy and immunofluorescence. CFP-CD63^+^ vesicles polarized toward the IS in P6 PKCδ-interfered cells expressing high levels of GFP-PKCδ (i.e., Supplementary Video 2, lower right panel), while very low expression levels of GFP-PKCδ did not restore MVB polarization when analyzed at the single cell level (i.e., Supplementary Video 2, lower left panel). More extensive end point analyses in fixed synapses were performed in C3 control and P6 PKCδ-interfered clones expressing high levels of GFP-PKCδ, and C9 control and S4 PKCδ-interfered clones expressing low levels GFP-PKCδ (Figure 2A). When we determined the percentage of synapses with polarized MVB we found that MVB polarization was restored in P6 PKCδ-interfered, GFP-PKCδWT-transfected clone to the levels observed for the C3 control clone (Figures 2B,C, lower panel). Similar results were obtained when low levels of GFP-PKCδWT were expressed in S4 PKCδ-interfered clone (Figure 2C, upper panel). In contrast, the expression of a kinase-dead, a dominant-negative PKCδ mutant (GFP-PKCδKD) (Figure 2D, upper panel), inhibited MVB polarization in the C3 control clone, and did not reestablish the polarization of the P6 PKCδ-interfered clone to the levels obtained in the C3 control clone (Figure 2D, lower panel). Thus, PKCδ kinase activity appears to be necessary for the positive effect of PKCδ on MVB polarization. In addition, we observed that GFP-PKCδ underwent activation upon IS formation, as assessed by WB analysis of T505 phosphorylation (3–4 fold induction) at the activation loop of GFP-PKCδ (Figure 2E) (34), in agreement with PKCδ activation described in mouse CTL (28). Altogether, these data indicate that PKCδ is required for the polarization of both MVB and the MTOC toward the IS in T lymphocytes.

### PKCδ Regulates Exosome Secretion

**Figure 3:**
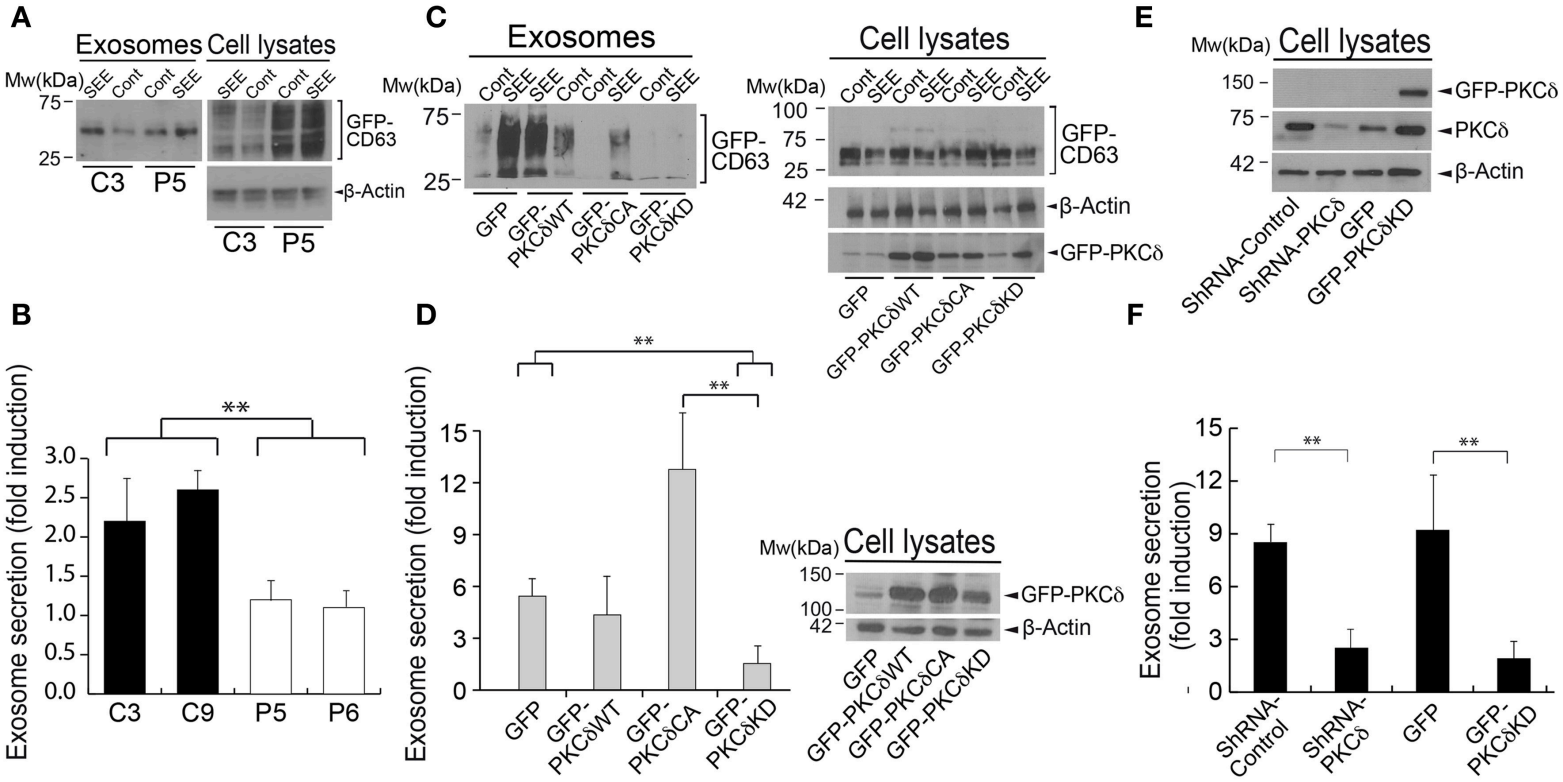
PKCδ regulates polarized exosome secretion. **(A)** GFP-CD63-expressing C3 control and P5 PKCδ-interfered Jurkat clones were challenged with either untreated (Cont) or SEE-pulsed (SEE) Raji cells for 6 h to induce synaptic conjugate formation and subsequent secretion of GFP-CD63^+^ exosomes. Exosomes were isolated, quantified by WB analysis of the GFP-CD63 reporter (using anti-CD63 Ab) (left panel) and normalized for cell number and cellular GFP-CD63 content in the corresponding cell lysates (right panel). **(B)** GFP-CD63-expressing C3 and C9 control, P5 and P6 PKCδ-interfered Jurkat clones were challenged with either non-pulsed (Cont) or SEE-pulsed (SEE) Raji cells for 6 h to induce synaptic conjugate formation and subsequent secretion of GFP-CD63^+^ exosomes. Exosomes were isolated and quantified by WB as in **(A)**. Data are means of normalized exosome secretion expressed as *x-*fold induction with respect to the non-stimulated control plus SD obtained from several experiments as described in **(A)** (*n* = 3), and single-factor ANOVA was performed between the indicated groups. ^**^p ≤ 0.05. **(C)** C3 control clone that co-expressed GFP-CD63 together with GFP, GFP-PKCδWT, GFP-PKCδCA, or GFP-PKCδKD was challenged as described above and exosomes were isolated. WB analysis of the GFP-CD63 reporter in exosomes (left panel) and cellular GFP-CD63 and β-actin (right panel) for normalization was performed as in **(A)**. In addition, WB of cell lysates was developed with anti-ratPKCδ to analyze the expression of the different PKCδ mutants in all conditions after the synaptic challenge (± SEE). **(D)** In the left panel, data are means of normalized exosome secretion expressed as *x-*fold induction plus SD from several experiments as described in **(C)** (*n* = 3). In the right panel, cell lysates were immunoblotted with anti-ratPKCδ to compare the levels of the different GFP-PKCδ constructs before the synaptic challenge. A non-specific band with an apparent mobility near the GFP-PKCδ chimeras was observed in lysates from cells co-transfected with GFP. **(E)** Human primary T-lymphoblasts were transduced by nucleofection with GFP-CD63 and the indicated interference or expression plasmids, and subsequently analyzed for endogenous PKCδ expression or ectopic GFP-mouse-PKCδ expression. **(F)** Subsequently, cells were stimulated with plastic-bound anti-TCR and exosome secretion was analyzed as in **(A)**. Data are means of normalized exosome secretion expressed as *x-*fold induction with respect to the non-stimulated control plus SD obtained from several experiments (*n* = 3); and single-factor ANOVA was performed between the indicated groups. ^**^p ≤ 0.05.

Since the polarization and degranulation of MVB at the IS are necessary for exosome secretion and apoptosis induction in T lymphocytes (13, 14, 25), we next studied the consequences of deficient MVB polarization on exosome secretion by several approaches. For the first approach, control and PKCδ-interfered Jurkat clones expressing the MVB/exosome reporter GFP-CD63 were challenged with untreated (control) or SEE-pulsed Raji cells (SEE). After 6 h of stimulation, exosomes were purified from the cell culture supernatants and quantified by WB analysis of GFP-CD63 (26). This allowed us, on one hand, to enhance the sensitivity of the assay (25) and, on the other hand, to exclusively quantitate and normalize the exosomes secreted by the Jurkat clones, avoiding the exosomes secreted by the Raji cells. We found that normalized inducible exosome secretion was decreased in P5 and P6 PKCδ-interfered clones when compared with C3 and C9 control clones (Figures 3A,B). Second, we transiently co-expressed in the C3 control clone the exosome reporter plasmid GFP-CD63 together with different GFP-PKCδ versions or GFP, as a control, and challenged them with SEE-pulsed Raji cells. WB analysis showed similar levels of expression of the GFP-PKCδ chimeras before the synaptic challenge (Figure 3D, right panel) and somewhat variable after the challenge (Figure 3C, right panel). The expression of a dominant-negative, kinase-dead PKCδ mutant (GFP-PKCδKD) strongly reduced the normalized secretion of exosomes induced upon IS formation (Figures 3C,D), in agreement with the data obtained in PKCδ-interfered clones (Figure 3B). By contrast, expression of a constitutively active PKCδ mutant (GFP-PKCδCA) strongly enhanced exosome secretion induced after IS formation (Figures 3C,D). Third, we stimulated control and PKCδ-interfered Jurkat clones with anti-TCR and, subsequently, we measured the number of secreted exosomes by nanoparticle tracking analysis (NTA). We observed a significant reduction in the concentration of exosomes (particles/ml) secreted upon TCR stimulation in the P5 PKCδ-interfered clone when compared with the C3 control clone (Supplementary Figure 3). However, the size distribution of the exosomes was not affected by PKCδ interference (Supplementary Figure 3). Next, we extended the results obtained in the Jurkat cells to human primary T lymphoblasts. As shown in Figure 3, both the interference in PKCδ expression and the transient expression of GFP-PKCδKD mutant (Figure 3E) inhibited TCR-stimulated exosome secretion (Figure 3F). Together, these results demonstrate that PKCδ is necessary for inducible exosome secretion in T lymphocytes.

### PKCδ Regulates AICD

**Figure 4:**
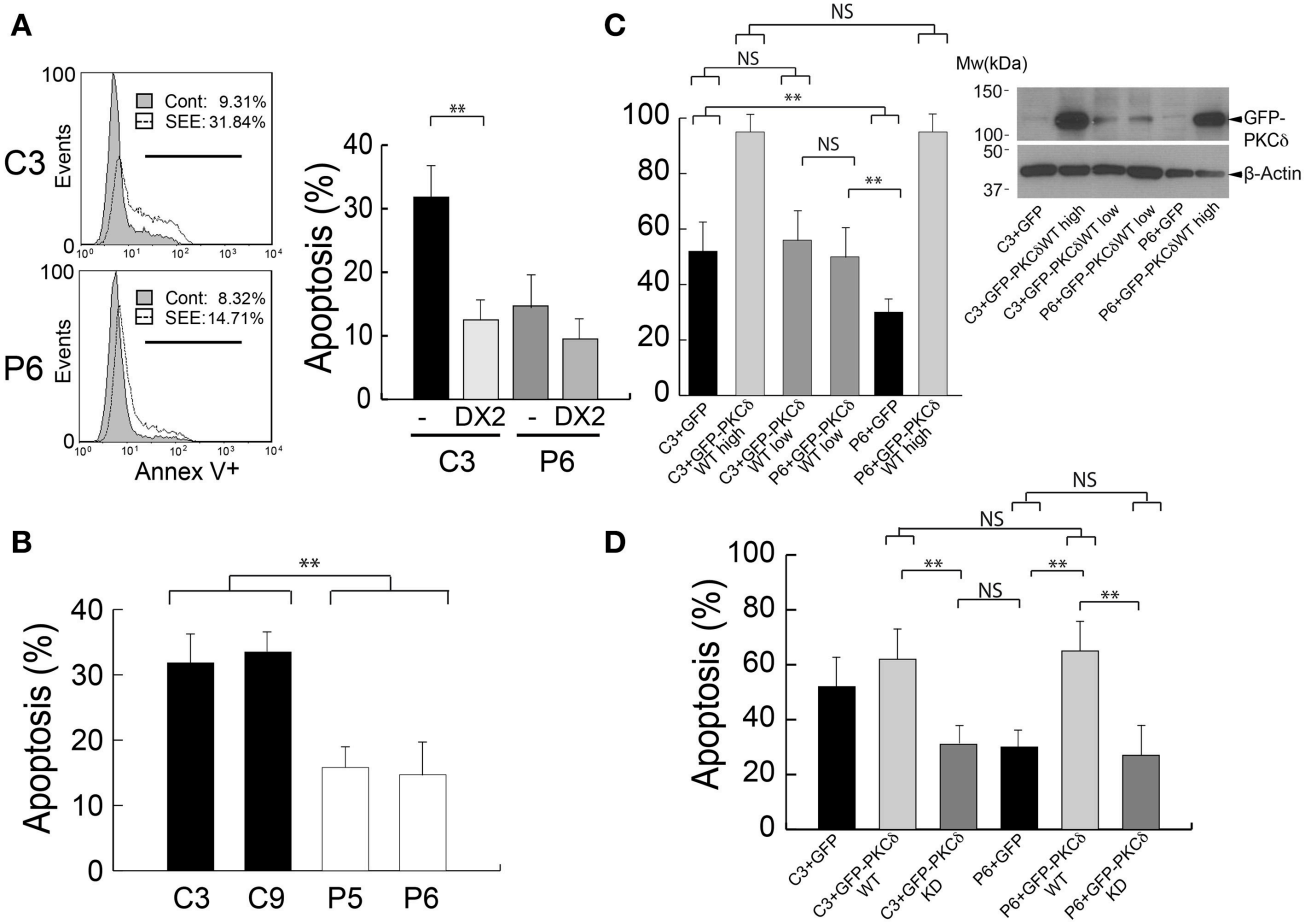
PKCδ regulates AICD. **(A)** Left panel, C3 control and P6 PKCδ-interfered Jurkat clones were challenged either with CMAC-labeled non-pulsed (Cont) or SEE-pulsed (SEE) Raji cells for 6 h, and the percentage of Annexin-V^+^ apoptotic cells (line marker) was determined by flow cytometry analysis after gating to exclude CMAC^+^ Raji cells. Right panel, same as left panel, but the Jurkat clones were preincubated or not (–) with blocking anti-Fas antibody (DX2) before the challenge with SEE-pulsed Raji cells. Data are means of the percentage of apoptosis plus SD obtained from several experiments (*n* = 3). **(B)** C3, C9 (control) and P5, P6 (PKCδ-interfered) Jurkat clones were challenged as in **(A)**, and the percentage of Annexin-V^+^ apoptotic cells was determined by flow cytometry analysis after gating to exclude CMAC^+^ Raji cells. Data are means of the percentage of apoptosis plus SD obtained from several experiments (*n* = 3). **(C)** Left panel, C3 control and PKCδ-interfered P6 Jurkat clones transfected with GFP or different amounts of GFP-PKCδWT expression plasmids (high = 30 μg, low = 10 μg) were challenged with CMAC-labeled, SEE-pulsed Raji cells for 12 h, and apoptosis induction was assessed in the transfected cells as shown in **(B)**. Right panel, WB analysis of GFP-PKCδ expression in cells used in the experiment from the left panel. **(D)** C3 control and P6 PKCδ-interfered Jurkat clones were transfected with the indicated expression plasmids and were challenged with CMAC-labeled, SEE-pulsed Raji cells for 12 h and apoptosis induction in the transfected cells was assessed as shown in **(B,C)**. Data are means plus SD (*n* = 3). Single-factor ANOVA was performed between the indicated groups. NS, not significant; ^**^p ≤ 0.05.

FasL-containing exosomes secreted upon IS formation are involved in autocrine T lymphocyte AICD (13, 14). Thus, it is conceivable that the decreased exosome secretion observed in PKCδ-interfered clones may affect AICD. To test this hypothesis, control and PKCδ-interfered Jurkat clones were challenged with non-pulsed or SEE-pulsed Raji cells for 6 h and end point apoptosis was measured by Annexin-V binding and flow cytometry analysis. We observed a 3.4-fold increase in apoptosis in the C3 control clone (9% apoptotic cells without SEE vs. 32% apoptotic cells with SEE). However, the apoptosis induction in P6 PKCδ-interfered clone was lower, with a 1.8-fold increase (8% apoptotic cells without SEE vs. 15% apoptotic cells with SEE) (Figure 4A, left panel). Comparable results were obtained when other control and PKCδ-interfered clones were challenged in parallel (C9 and P5, Figure 4B). The AICD produced upon synaptic challenge was dependent of Fas ligand/Fas interaction, since apoptosis was inhibited when C3 and P6 clones were pre-incubated with a blocking antibody directed against human Fas (clone DX2) (13) before the challenge with SEE-pulsed Raji cells (Figure 4A, right panel). In addition, time-lapse imaging of GFP-PKCδ-expressing clones showed evidence for early apoptosis (i.e. plasma membrane blebs and cell shrinkage) between 2 and 5 h after IS formation (Supplementary Video 3, red arrows), in agreement with our previous results (25). Furthermore, expression of both high and low levels of GFP-PKCδ in the C3 control and P6 PKCδ-interfered clones (Figure 4C, right panel) showed a similar percentage of apoptotic cells upon stable IS formation (Supplementary Video 3), as assessed by flow cytometry (Figure 4C). Thus, deficient apoptosis was rescued when GFP-PKCδWT was expressed in P6 PKCδ-interfered Jurkat clone, in accordance with the observed increase in MVB polarization toward the IS (Figure 2C). However, the expression of a kinase-dead PKCδ mutant (GFP-PKCδKD) in the P6 PKCδ-interfered clone did not restore the AICD to the levels obtained in the C3 control clone (Figure 4D). Thus, PKCδ kinase activity seems to be required for the positive effect of PKCδ on AICD as it was for MVB polarization and exosome secretion. Together these data indicate that PKCδ is required for the secretion of FasL-containing exosomes and subsequently AICD.

### DAG Recruits PKCδ to Polarized MVB but Not to the IS

**Figure 5:**
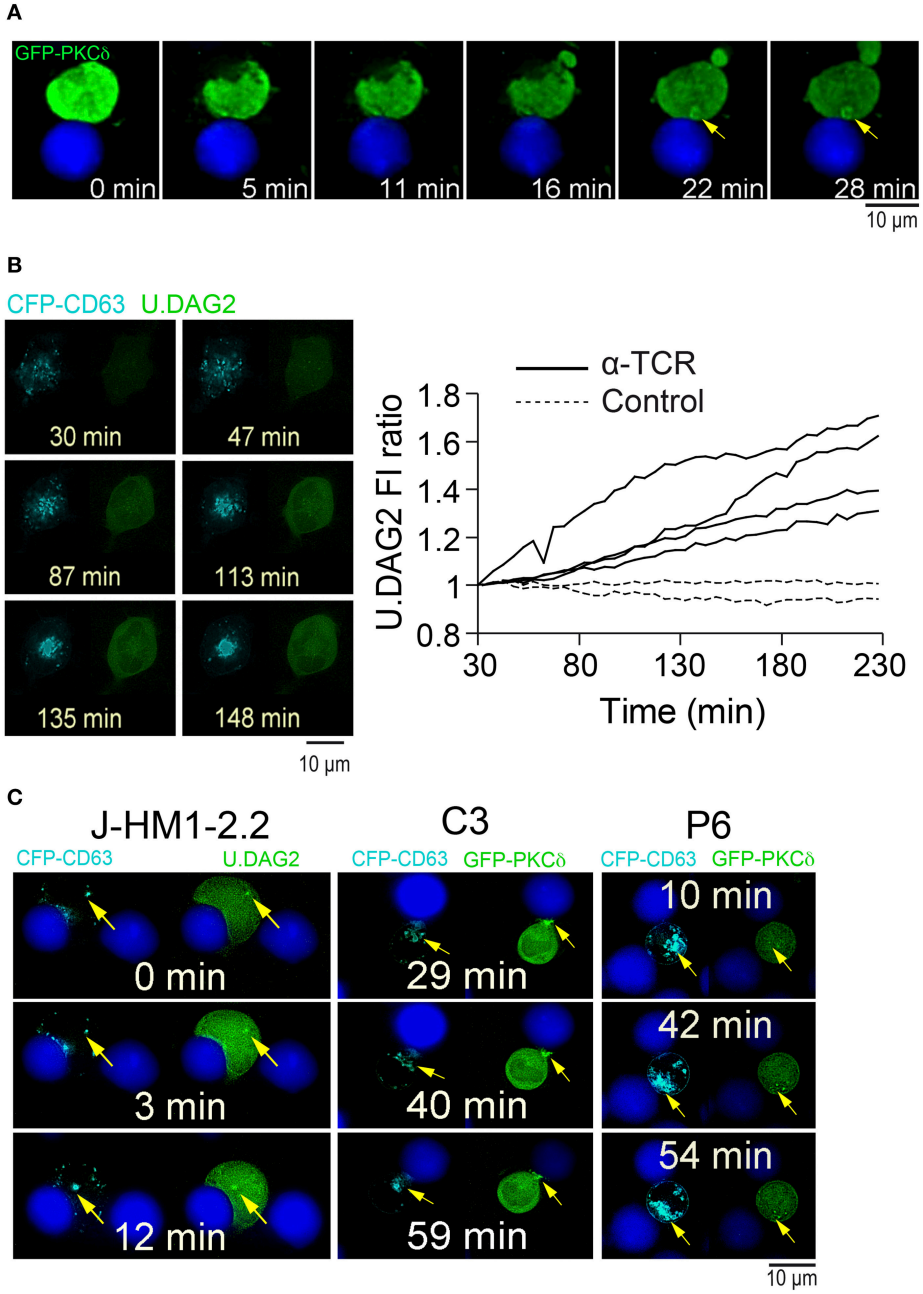
Subcellular localization of GFP-PKCδ and U.DAG2 upon activation. **(A)** C3 control Jurkat clone expressing GFP-PKCδWT was challenged with CMAC-labeled, SEE-pulsed Raji cells and the synaptic conjugates imaged by time-lapse fluorescence microscopy. Relevant frames corresponding to Supplementary Video 2 (top right panel) are shown. The yellow arrow indicates accumulation of GFP-PKCδ **(B)** J-HM1-2.2 cells co-expressing CFP-CD63 and U.DAG2 were untreated (Control) or challenged with plate-bound anti-TCR (α-TCR), imaged by time-lapse fluorescence microscopy, and the U.DAG2 FI ratio was calculated. Left panels show relevant frames of two fluorescence channels corresponding to the 2nd time-lapse video from concatenated (Supplementary Video 4) (TCR stimulation). Right panel, U.DAG2 FI ratio was calculated as the average FI corresponding to each cell ROI at different times relative to the average FI at *t* = 30 min (FI at the indicated time point/FI at *t* = 30 min) and corresponded to untreated cells (*n* = 2, discontinuous lines) and cells challenged with plate-bound anti-TCR (*n* = 4, continuous lines). **(C)** J-HM1-2.2 cells co-expressing CFP-CD63 and U.DAG2 (left panels), and C3 control and P6 PKCδ-interfered Jurkat clones co-expressing CFP-CD63 and GFP-PKCδ (right panels) were challenged with CMAC-labeled, SEE-pulsed Raji cells and imaged by time-lapse microscopy. Relevant frames corresponding to J-HM1-2.2 cells (Supplementary Video 5), C3 control clone (Supplementary Video 2, top left panel) and P6 PKCδ-interfered clone (Supplementary Video 2, lower right panel) are shown. The yellow arrow indicates the accumulations of MVB, U.DAG2 and GFP-PKCδ. CMAC labeling of Raji cells in blue, CFP-CD63 in cyan and U.DAG2 or GFP-PKCδ in green. Scale bars, 10 μm.

**Figure 6:**
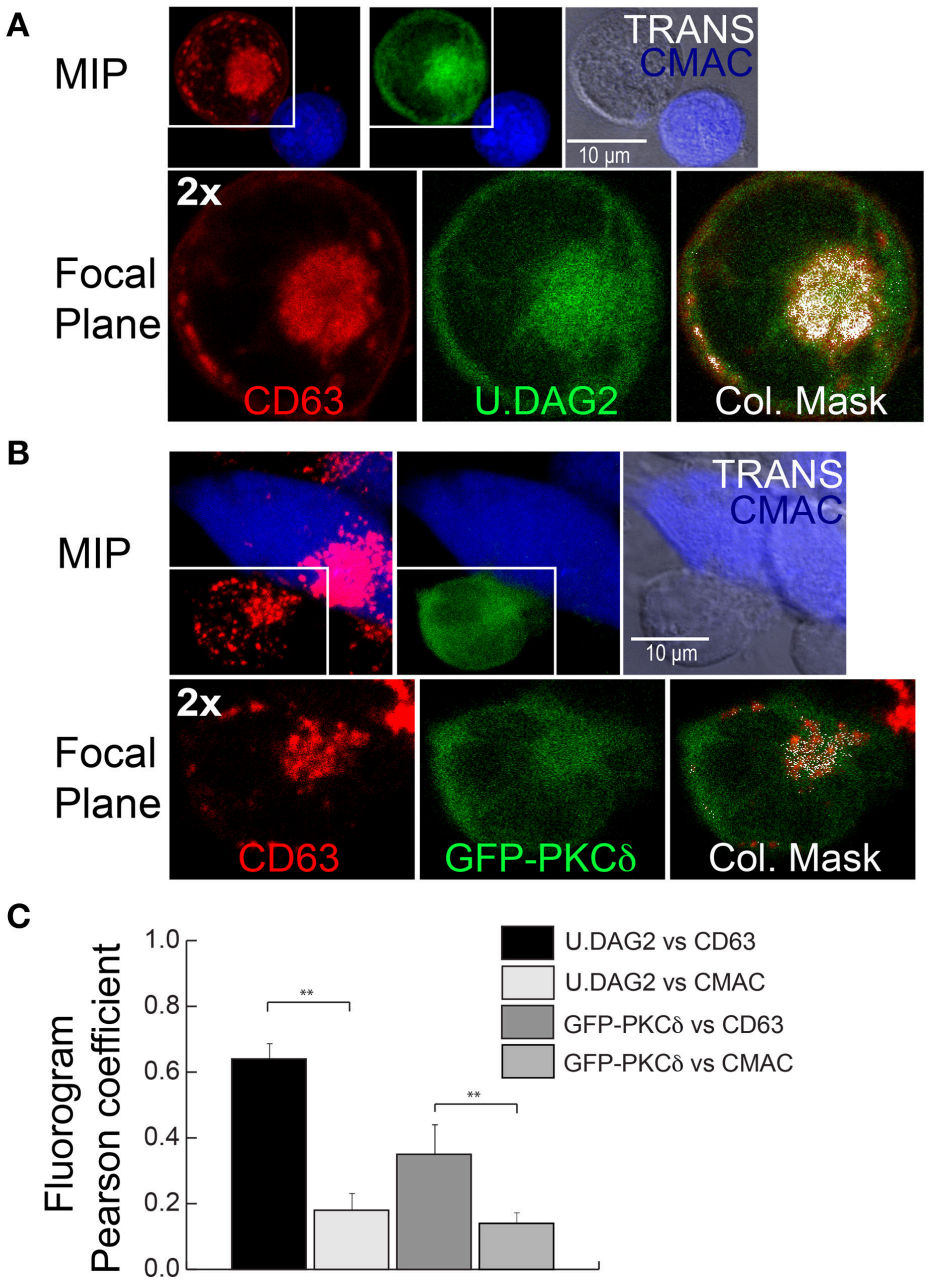
Colocalization analysis of GFP-PKCδ, U.DAG2 and CD63. **(A)** J-HM1-2.2 cells expressing U.DAG2 were challenged with CMAC-labeled SEE-pulsed Raji cells. After 1 h of conjugate formation, fixed cells were immunolabeled with anti-CD63 Ab to visualize the MVB and imaged by confocal microscopy. Maximal Intensity Projection (MIP) (top row), representative single focal plane (*Z* = 7, 2x zoom, bottom row) and colocalization mask of the same field are shown. The right-hand bottom panel shows the merged images with structures containing both CD63 and U.DAG2 appearing white (Pearson's correlation coefficient of fluorogram = 0.69). **(B)** P6 PKCδ-interfered clone expressing GFP-PKCδ was challenged with CMAC-labeled SEE-pulsed Raji cells. After 1 h, fixed cells were immunolabeled and imaged as in **(A)** MIP (top row), representative single focal plane (*Z* = 5, 2x zoom, bottom row) and colocalization mask (Pearson's correlation coefficient of fluorogram = 0.26) of the same field are shown. CMAC labeling of Raji cells in blue, CD63 in red and U.DAG2 or GFP-PKCδ in green. Scale bars, 10 μm. **(C)** C3 control clone expressing U.DAG2 or GFP-PKCδ were challenged as in **(A)** for 1 h, fixed and immunolabeled with anti-CD63. In control experiments developed in parallel, Jurkat cells were preloaded with CMAC, and the respective correlation coefficients of GFP-PKCδ and U.DAG2 with respect to cytosolic CMAC (as a negative control for colocalization analyses) were analyzed. Immuno-labeled cells were imaged by confocal microscopy and pairwise, Pearson's correlation coefficient of scatter-plot (fluorogram) between the indicated channels was calculated as indicated in Material and Methods. Results are expressed as means plus SD (*n* = 3) analyzing at least 20 synapses per experiment. Single-factor ANOVA was performed between the indicated groups. ^**^p ≤ 0.05.

To improve our understanding of the molecular basis underlying the PKCδ effect on MVB polarization, we first analyzed the dynamics of PKCδ subcellular localization upon IS formation. Imaging of control Jurkat clones expressing GFP-PKCδ showed that IS formation induced a partial redistribution of GFP-PKCδ from cytosol to a ring-shaped area nearby the MVB and proximal to the IS that was concomitant to its activation (Supplementary Video 2 -top right panel-, Figures 2B, 5). It has been described that DAG recruits PKCδ to membranes through its C1 DAG-binding domains, and this leads to PKCδ activation (49). Thus, in order to optimally detect subcellular changes in the PKCδ activator DAG, we used U.DAG2, which upon DAG binding increases its fluorescence (35, 36). We first validated the sensor transfecting J-HM1-2.2 cells with U.DAG2 and stimulated them with either CCH or PMA. We used GFP-C1bPKCθ, another DAG sensor based on the C1b domain of PKCθ, as a control since it does not enhance its fluorescence when it binds DAG (50). The FI ratio along time for each construction was calculated as the average FI corresponding to the cell ROI at different time points relative to the average FI of the same cell ROI at the initial time point. CCH and PMA induced recruitment of both DAG sensors to the plasma membrane (although the effect with PMA was stronger than CCH), and a concomitant increase in relative U.DAG2 FI, as opposed to relative GFP-C1bPKCθ FI (Supplementary Figure 4 and Supplementary Video 4, first concatenated video). We then co-expressed CFP-CD63 and U.DAG2 in J-HM1-2.2 cells and stimulated them with either plastic-bound anti-TCR (51) or with CMAC-labeled SEE-pulsed Raji cells to test whether DAG was generated during MVB polarization. Time-lapse imaging showed an increase in the U.DAG2 FI ratio (Figure 5B) upon TCR stimulation, concomitant with the centripetal convergence of MVB toward the center of the cell in contact with the coverslip surface, that corresponded to the secretory domain next to cSMAC (52) (Supplementary Video 4, 2nd concatenated video, Figure 5B). Furthermore, during IS formation, accumulations of U.DAG2 co-migrating with MVB and a simultaneous enhancement of U.DAG2 FI, were also observed (yellow arrows in Figure 5C, Supplementary Video 5 and Supplementary Figure 6). Interestingly, when we co-expressed CFP-CD63 and GFP-PKCδ, we observed GFP-PKCδ accumulations that may co-migrate with MVB toward the IS (yellow arrows in J-HM1-2.2 and P6 panels, Figure 5C and Supplementary Video 2), although temporal segregation between these subcellular structures was also evident (yellow arrows in C3 panel, Figure 5C and Supplementary Video 2). To further study this potential association and to analyze the subcellular localization of U.DAG2 and GFP-PKCδ with respect to the MVB, we carried out confocal microscopy. We found that the accumulation of U.DAG2 observed in living cells upon IS formation colocalized with the MVB marker CD63 (Figure 6A). Furthermore, GFP-PKCδ also partially colocalized with CD63+ structures (Figure 6B). These partial colocalizations of U.DAG2 and GFP-PKCδ with CD63 were indeed specific, since Pearson's correlation coefficients of scatter plot fluorograms were significantly different from those corresponding to the analysis of U.DAG2 or GFP-PKCδ vs. cytosolic CMAC, (Figure 6C). Remarkably, we could not detect any recruitment of GFP-PKCδ to the synaptic membrane (Supplementary Video 6) whereas, in parallel, relocalization of PKD1 or C1bPKCθ domain to this membrane was evident (Supplementary Videos 6, 7). The relocalization of both PKD1 and C1bPKCθ was dependent on transient DAG production at the IS, since interference in DGKα that enhances both DAG levels (24) and PKD1 activation (26), increased PKD1 and C1bPKCθ residence half-life at the synapse as assessed by time-lapse video analysis (Supplementary Video 7 and Supplementary Figure 5). DGKα attenuation significantly enhanced mean DsRed2-PKD1 residence half-life at the synaptic membrane from 15–20 up to 40 min and GFP-C1bPKCθ half-life from 40 up to 75 min (Supplementary Figure 5). Together, these results suggest that upon synaptic activation DAG species, capable of recruiting a portion of PKCδ to endomembranes including MVB but not the IS, are produced.

### PKCδ Regulates the Spatiotemporal Reorganization of Cortical Actin at the IS

**Figure 7:**
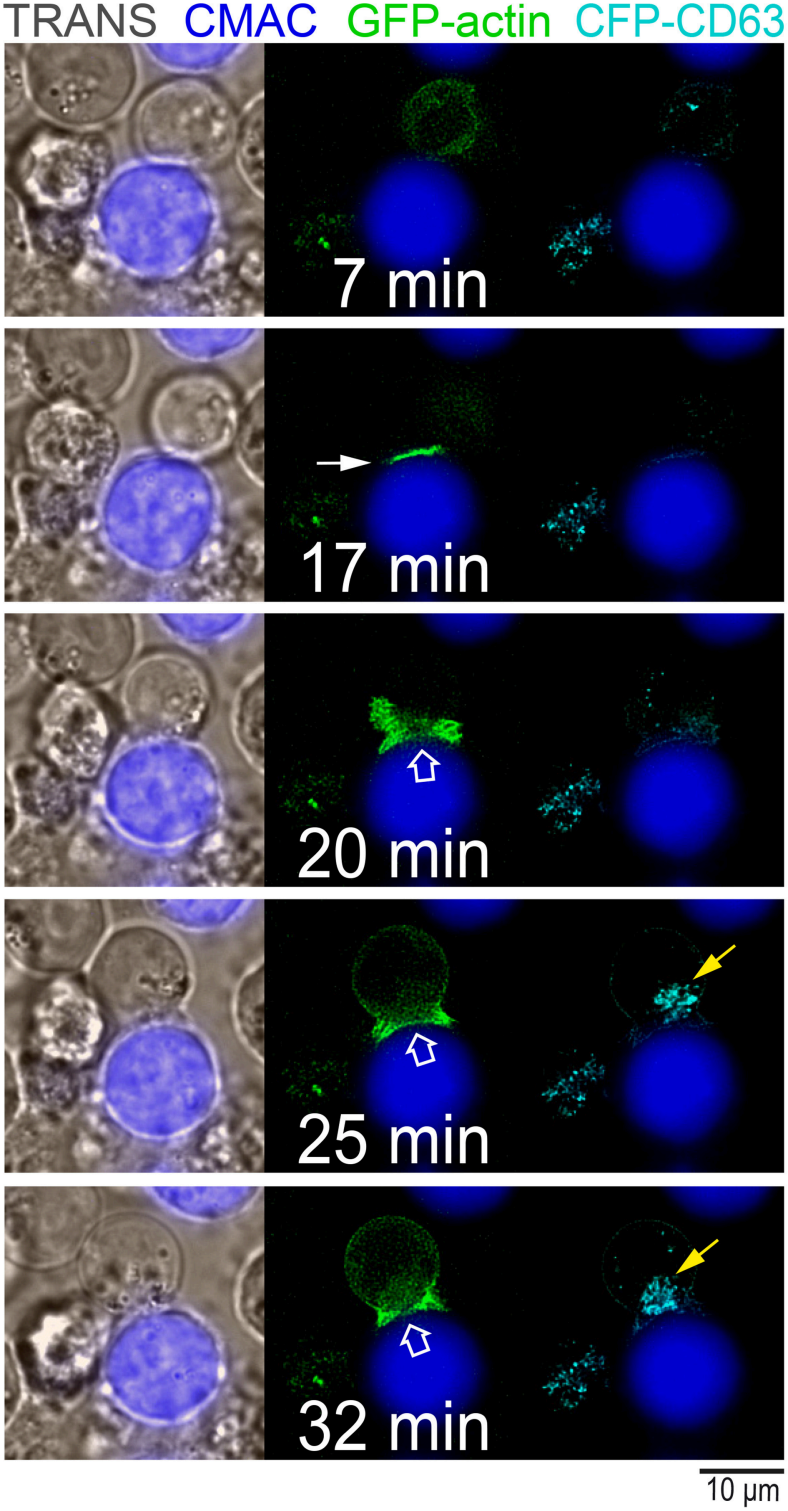
Reorganization of cortical actin and polarization of MVB at the IS. C3 control clone co-expressing GFP-actin and CFP-CD63 was challenged with CMAC-labeled, SEE-pulsed Raji cells and imaged by time-lapse fluorescence microscopy. Some representative frames from Supplementary Video 8 are shown. Left panel, transmittance and CMAC channels; middle panel, GFP-actin and CMAC channels; right panel, CFP-CD63 and CMAC channels. The initial burst of actin reorganization at the IS occurring upon synaptic contact (closed white arrow), the cortical actin depletion at the cIS (open white arrow) and the polarization of MVB (yellow arrow) toward the IS are indicated. CMAC labeling of Raji cells in blue, CFP-CD63 in cyan and GFP-actin in green. Scale bars, 10 μm.

**Figure 8:**
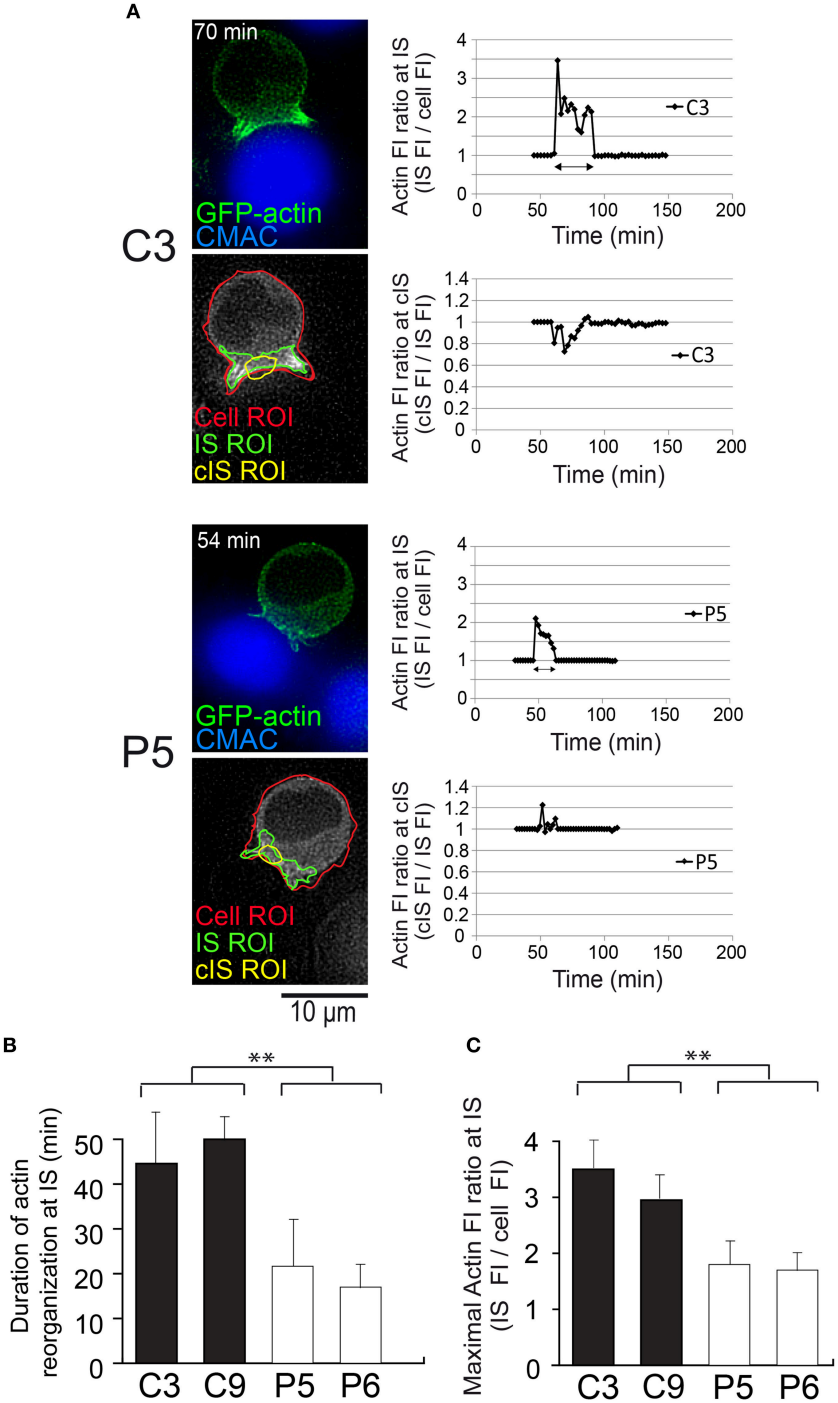
Kinetic analysis of cortical actin reorganization at the IS in PKCδ-interfered cells. C3, C9 (control) and P5, P6 (PKCδ-interfered) clones expressing GFP-actin were challenged with CMAC-labeled SEE-pulsed Raji cells and imaged by time-lapse fluorescence microscopy. **(A)** Kinetic image analysis to evaluate actin FI ratio at the synapse (IS) and at the central synapse (cIS) using the indicated ROIs (cell ROI, red line; synapse ROI, green line; central synapse ROI, yellow line) for C3 and P5 clones asynchronously developing synapses. Representative frames from Supplementary Video 9 at the indicated times after the addition of clones to the SEE-pulsed Raji cells and below the corresponding, superimposed ROIs, are depicted (left panels) as a reference. Kinetic analyses of the relative cortical actin FI at the IS (IS FI/cell FI) and at the cIS (cIS FI/IS FI) are shown (right graphs) for C3 (upper graphs) and P5 (lower graphs). The time t = 0 of the X axis scale corresponds to the addition of clones to the SEE-pulsed Raji cells, which occurred 45 min (for C3) and 30 min (for P5) before the beginning of the time-lapse capture (thick lines in the graphs) of Supplementary Video 9. The beginning of conjugate formation, which corresponds to the peak of actin FI ratio at the IS, occurred at 62 min (17 plus 45 min) for the C3 clone and 46 min (16 plus 30 min) for the P5 clone after the addition of clones to the SEE-pulsed Raji cells. Double-headed dark arrows correspond to the time intervals during which actin ratio values were different to 1 (i.e., length of the actin reorganization period). CMAC labeling of Raji cells in blue and GFP-actin in green. Scale bars, 10 μm. **(B)** Results are expressed as average duration of the interval of actin reorganization for C3, C9 (control) and P5, P6 (PKCδ-interfered) clones. **(C)** Same as **(B)** but results are expressed as average maximal actin FI ratio at the IS. Data are means plus SD (*n* = 3, analyzing at least 12 synapses from several different microscopy fields per experiment). Single-factor ANOVA was performed between the indicated groups. ^**^p ≤ 0.05. This figure is related to Supplementary Video 9.

It has been reported that the polarizations of the MTOC and cytotoxic granules toward the IS are conducted by a transient increase in cortical actin reorganization at the IS (4). This burst in cortical actin at the IS is followed by the clearance, and subsequent recovery, of cortical actin density at cIS. These events appear to be required for lytic granule and cytokine secretion in CTL and CD4^+^ lymphocytes, respectively (4, 5, 53). Thus, we studied whether actin reorganization at the IS occurs during traffic of MVB in T lymphocytes and whether PKCδ regulates this reorganization. To perform this analysis and to correlate actin reorganization at the IS with MVB polarization, we challenged control and PKCδ-interfered Jurkat clones, co-expressing GFP-actin and CFP-CD63, with SEE-pulsed Raji cells, and analyzed the changes of relative actin fluorescence intensity (FI) at the IS and at the cIS by time-lapse microscopy (Figures 7, 8; Supplementary Videos 8, 9). In actin reorganization kinetic experiments (Figure 8A), the actin FI ratio was calculated as the average actin FI corresponding to each subcellular ROI (synapse or central synapse regions) relative to the average FI of the indicated ROI (cell or synapse) at the same time point. In the C3 control clone, we observed a transient accumulation of cortical actin at the IS (closed white arrow, Figure 7), followed by the temporary depletion of actin at the cIS (open white arrows, Figure 7) and, almost simultaneously, the convergence and polarization of the initially scattered MVB toward the F-actin-low region at the cIS (yellow arrows in Figure 7 and Supplementary Video 8). Next, we analyzed the kinetics of the accumulation of cortical actin at the IS, defined as the time period showing a relative actin FI at the synapse (IS FI/cell FI) >1 (Supplementary Video 9 and Figure 8, first and third rows). In the left side of Figure 8A, some representative frames from Supplementary Video 9 at the indicated times after the addition of clones to the SEE-pulsed Raji cells and, below, the superimposed ROIs (cell, IS and cIS) used in these analyses are depicted. The C3 and C9 control clones showed significantly longer actin accumulation at the IS than the P5 and P6 PKCδ-interfered clones (Figure 8B, Supplementary Video 9). In addition, a difference in the magnitude of the F-actin reorganization process (measured as the maximal relative actin FI at the synapse) between the control and the PKCδ-interfered clones was observed (Figure 8A, right graphs, and Figure 8C). Moreover, no depletion of cortical actin at the cIS was observed in the P5 PKCδ-interfered clone, since the relative value of actin FI in the cIS (cIS FI/IS FI) remained >1 (Figure 8A, fourth row, Supplementary Video 9), while a transient actin depletion at cIS (cIS FI/IS FI remained <1) was observed in the C3 control clone (Figure 8A, second row).

**Figure 9:**
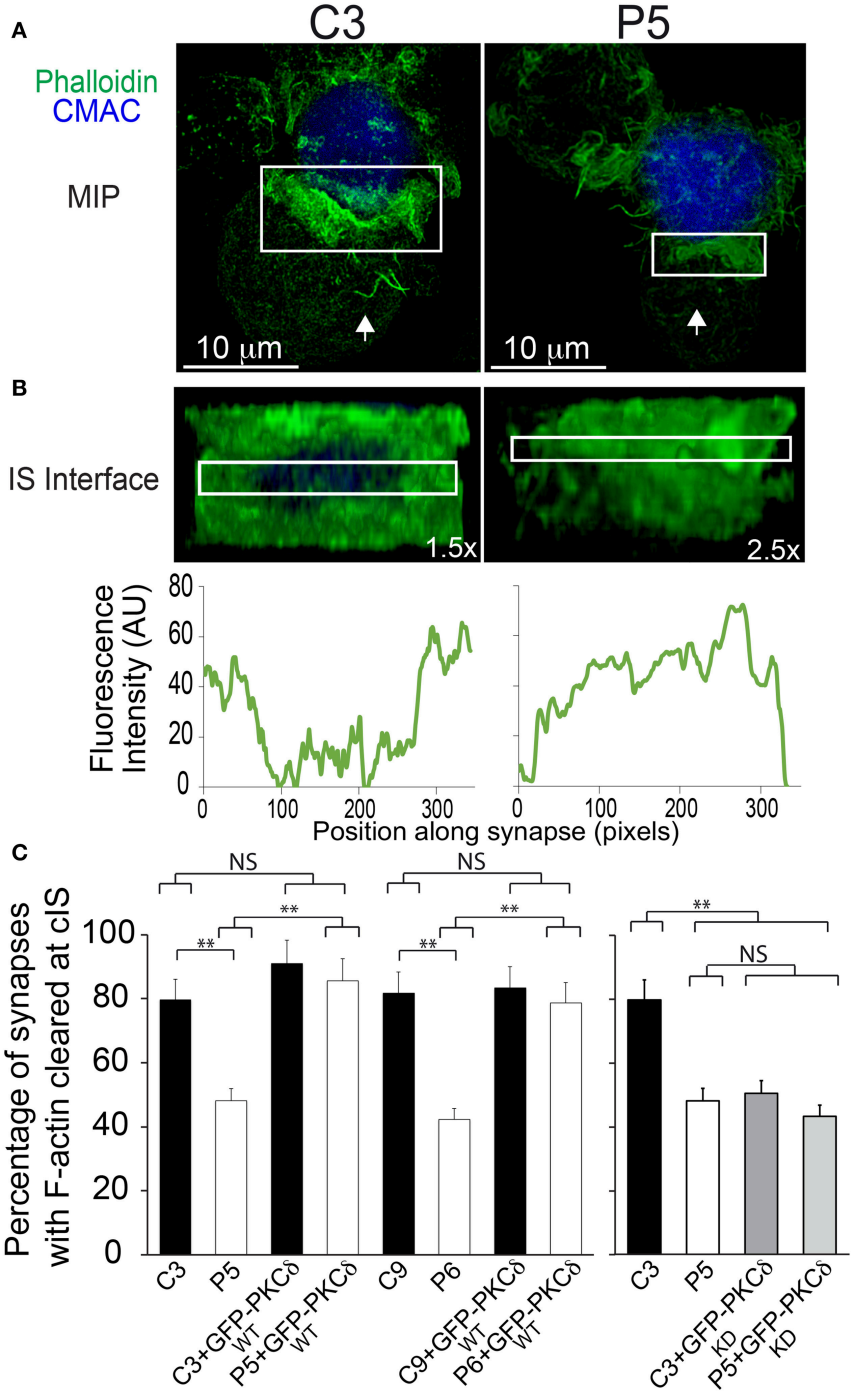
PKCδ regulates the spatial organization of cortical actin at the IS. C3 control and P5 PKCδ-interfered clones were challenged with CMAC-labeled SEE-pulsed Raji cells for 1 h, fixed, stained with phalloidinAF488 and imaged by confocal fluorescence microscopy. **(A)** Top views correspond to the Maximal Intensity Projection (MIP) of the indicated, two merged channels, in a representative example. White arrows indicate the direction to visualize the face on views of the synapse (IS interface) enclosed by the boxed ROIs (white rectangles) as shown in Supplementary Video 10. **(B)** Face on views of the IS. The enlarged ROIs from **(A)** (1.5x and 2.5x zoom, respectively) were used to generate (as shown in Supplementary Video 10) the IS interface images shown in the upper panels. The IS interface images of the phalloidin channel correspond to frame no. 44 of Supplementary Video 10. The plot profile analysis of phalloidin FI for this frame along the indicated rectangular ROIs is shown in the lower diagrams. CMAC labeling of Raji cells in blue and phalloidin in green. Scale bars, 10 μm. **(C)** Left panel, C3, C9 (control) and P5, P6 (PKCδ-interfered) clones were untransfected or transfected with GFP-PKCδWT or GFP-PKCδKD. Subsequently the clones were challenged with CMAC-labeled SEE-pulsed Raji cells for 1 h, fixed, immunolabeled with phalloidin AF546 and imaged by fluorescence microscopy. Subsequently, the face on views of synapses were generated as in **(B)** and the percentage of synapses with an F-actin depleted region at the central IS for the different cellular groups was calculated as described in Supplementary Figure 7. Data are means plus SD (*n* = 3) analyzing at least 54 synapses per experiment. Single-factor ANOVA was performed between the indicated groups. ^**^p ≤ 0.05.

Since 2D (X, Y) time-lapse analysis did not provide spatial information of the synaptic actin architecture, we analyzed 3D distribution of F-actin at the IS in confocal microscopy image stacks. To perform these measurements, we generated 2D projections of the IS interface of both control and PKCδ-interfered clones, labeled with phalloidin (Supplementary Video 10 and Figure 9). The boundary of the T lymphocyte/APC synaptic contact was defined by the distal SMAC (dSMAC), which consists of a circular array of F-actin accumulation at the edge of the T cell/APC interface (6) (47) (Supplementary Figure 7B). Thus, the IS area was delimited by the edge of the F-actin signal. When necessary, the definition of the regions of interest (ROI) to measure the F-actin-low (Fact-low) cIS area and the IS area was performed by using automated algorithms as described in the Materials and Methods section. Next, these defined ROI areas (Fact-low cIS area and IS area) were measured, and the relative area of the F-actin depleted region at cIS (Fact-low cIS area/IS area) was determined (Supplementary Figures 7B,C). In addition, the intensity of F-actin along the IS interface was analyzed (Supplementary Video 10 and Figures 9A,B). We found a significant reduction in the percentage of synapses substantiating a F-actin-low region at cIS, with relative area > 0.1 in the P5 PKCδ-interfered cells when compared with C3 control cells (80%±6 F-actin depleted synapses in C3 clone vs. 48%±4 in P5 clone; Supplementary Figure 7C and Figure 9C). Similar results were obtained when C9 control and P6 (PKCδ-interfered) clones were compared (Figure 9C). In addition, the expression of GFP-PKCδWT both in the P5 and P6 PKCδ-interfered clones restored the percentage of synapses exhibiting the depletion of F-actin at cIS to the levels observed for the C3 and C9 control clones (i.e., 48% ± 4 in P5 clone vs. 85% ± 9 in P5 clone expressing GFP-PKCδ (Figure 9C). In contrast, the expression of a kinase-dead PKCδ mutant (GFP-PKCδKD) in P5, PKCδ-interfered clone, did not recover the depletion of F-actin at cIS to the levels obtained in C3 control clone (Figure 9C). Together these data indicate that PKCδ participates in the spatiotemporal reorganization of actin at the IS and the subsequent polarization of MVB toward the F-actin-low region at the cIS and exosome secretion.

## Discussion

In this work we have established that activation of TCR at the IS induces PKCδ activation, which is necessary for the polarization of MTOC and the polarized secretory traffic of MVB toward the IS and exosome secretion. Concomitantly, we demonstrate that PKCδ-interfered T lymphocytes are defective in Fas-FasL dependent AICD. Since exosomes containing FasL have been shown to be involved in AICD (13, 14), a key process in regulating T lymphocyte homeostasis (10), our data support a new role of PKCδ in exosome-controlled immune regulatory processes. Both FasL and Fas deficiencies are involved in the development of several autoimmune lymphoproliferative syndromes (ALPS) both in humans and mice (10, 54). In this context, human patients were identified with a homozygous loss-of-function mutation of PKCδ resulting in deficient PKCδ expression. These patients developed ALPS resembling the phenotype of PKCδ KO mice (55). Thus, PKCδ is important for the maintenance of T lymphocyte homeostasis as well as the FasL/Fas system. Regarding the functional consequences of defective exosome secretion occurring in PKCδ-interfered cells, it has been reported that exosomes contribute to thymic development (19). Negative selection in thymocytes, which is induced via TCR-controlled apoptosis (AICD), is considered an important mechanism regulating thymocyte development and immune tolerance (56). In addition, immature thymocytes from PKCδ KO mice were protected from apoptosis, indicating a clear pro-apoptotic role of PKCδ (57). It remains to be established whether the systemic autoimmune phenotype and lack of tolerance observed in PKCδ KO mice and human patients with autosomal recessive mutations in PKCδ (58) might be due to defective exosome secretion and subsequent AICD occurring during thymic development.

T and B lymphocytes are the only cells in which triggering of cell surface receptors such as TCR and BCR controls induce exosome secretion (16). Following their secretion, exosomes participate in several antigen-dependent, important immune functions (59). We have described how exosome secretion in Th lymphocytes follows TCR stimulation and IS formation (13, 25). Regarding the TCR-regulated secretory traffic of MVB, several reports have previously established the essential role of DAG and its negative regulator DGKα in the polarized traffic of MVB/late endosomes and lytic granule secretion (13, 20, 24, 25), but the molecular basis by which DAG regulates this traffic remained largely unknown. To address this important point, we have analyzed Jurkat-Raji synapses because of the closeness of this experimental system to the biological reality (T cell-APC synaptic conjugates). This analysis has used scanning confocal microscopy and time-lapse fluorescence microscopy combined with post-acquisition deconvolution, due to the enhanced signal-to-noise ratio of the images, high temporal resolution, and adaptability to the simultaneous acquisition of multiple fluorochromes in developing synapses (45). As a drawback, only those APC-T cell interfaces that were perpendicular to the plane of focus along the Z-axis could be properly analyzed (45). Since other experimental models that may facilitate image capture and analyses do not mimic the complex, irregular surface of an APC or a target cell, and may raise to non-physiological interactions in the IS (60–63), we consider the experimental system used here appropriate to address some biological events occurring at the IS. In addition, with the use of the PKCδ C1-based U.DAG2 sensor, we have shown that upon synaptic activation, DAG species are produced that may recruit PKCδ at endomembranes including MVB. The U.DAG2 sensor was described to produce rapid and robust changes in green fluorescence in a live-cell assay, and these changes were reversible, since fluorescence returns to baseline levels 20–30 s after stimuli removal (35). In addition, there was an additional fluorescence increase of the U.DAG2 sensor due to the conformational changes (35, 36); this was an additional reason to use the sensor in our effort to reveal potential subtle DAG changes that may occur. Thus, the sensor indeed appears to be sensitive enough, although we cannot fully exclude that some very transient or very small DAG changes may not be detectable by the sensor. In addition, we cannot entirely exclude that the PKCδC2 domain (absent in the U.DAG2 probe) may have an indirect, additional role on the selectivity of PKCδ for DAG species produced at the plasma membrane or endomembranes. In this context, we could not detect any U.DAG2 accumulation or PKCδ recruitment at the IS membrane, although DAG-mediated accumulation of C1bPKCθ or PKD1 at the IS was induced in parallel (Supplementary Videos 6, 7), as shown by other researchers (20, 50, 64). Thus, most probably, DAG species that were produced upon PLC activation by TCR were capable of recruiting PKCθ (65) or PKD1 (46) to the IS via their C1 domains, but not PKCδ (66). In addition, our results support that, also upon synaptic activation, distinct DAG species to those generated at the IS are produced into endomembranes (such as MVB), which are capable of recruiting and activate PKCδ. Supporting our data, PKCδ has been found in lytic granules form CTLs (28). In this context, it has been shown that differences in intrinsic affinities and selectivities among the C1 and C2 DAG-binding domains for different DAG species control the rate, magnitude, duration, and subcellular localization of the diverse PKC isotypes (67, 68). The data from other authors, based on the lack of recruitment of PKCδ to the IS (64), may apparently argue against a contribution of PKCδ to MTOC polarization. However, we propose that TCR signaling regulates the production of distinct DAG species at different subcellular locations (IS and MVB endomembranes), with each species recruiting different PKC isotypes, which may be together necessary for MTOC polarization. Indeed, this may conciliate these data with our results regarding the involvement of PKCδ in MTOC polarization toward the IS. Regarding the molecular bases that may underlie the PKCδ effect on F-actin reorganization, it has been described that phosphorylation of paxillin, an actin regulatory protein, by PKCδ regulates integrin-mediated adhesion and migration of lymphoid cells (69). In particular, PKCδ phosphorylates paxillin at T538, leading to the depolymerization of the actin cytoskeleton (69). In addition, paxillin phosphorylation is required for the degranulation of CTL (70). Thus, we are analyzing the phosphorylation of paxillin at T538 in control Jurkat clones. Our preliminary data suggest that both pharmacologic (PMA), anti-TCR stimulation and synaptic stimulation of Jurkat control clones induced a strong phosphorylation of paxillin at T538 (not shown). Further experiments are required to analyze whether PKCδ-interfered clones substantiate comparable paxillin phosphorylation upon stimulation. The results from these experiments may provide some clue to explain the PKCδ role on F-actin reorganization leading to MTOC polarization.

Here we have studied the role of cortical actin reorganization in the polarized traffic of MVB leading to exosome secretion at the IS in Th lymphocytes. PKCδ-interfered clones forming synapses exhibited quantitative alterations (duration and magnitude of actin reorganization) but also qualitative differences (absence of depletion of F-actin at the cIS) in actin rearrangement at the IS when compared with control clones. Any of these alterations acting alone or in combination, have been described to affect T-cell activation, polarized secretion, AICD and CTL effector functions [reviewed in (3)]. In fact, the phenotype we describe here resembles the alterations found in T lymphocytes deficient in TAGLN2 (71) or HS1 (51). Thus, the altered actin stabilization at the IS we found may underlie the deficient MVB polarization occurring in PKCδ-interfered clones. The initial increase in F-actin at the IS was followed by a decrease in F-actin density at the cIS, and this event appears to be the limiting step for exosome secretion. This process is similar to the degranulation of cytotoxic granules and cytokine-containing secretory granules at the F-actin-depleted region that contains a specialized secretory domain in CTL and Th lymphocytes (4, 5, 53). Interestingly, in both cell types cortical actin reorganization at the IS, followed by the polarization of MTOC and secretory granules toward the IS, was also reported (4, 47, 53, 72). Thus, these sequential events are essentially common to IS formed by CTL or Th cells, although both the nature and cargo of the secretory granules in these cells are quite different. In addition, CTL form much more transient synapses than Th cells, lasting only a few minutes, as the target cells are killed (2, 5). This is probably due to the fact that the optimal CTL function requires rapid and transient contact in order to deliver as many lethal hits as possible to several target cells, whereas stable, lengthy synapses (>20–30 min up to several hours) formed by Th cells, as we studied here, are necessary for both directional and continuous secretion of stimulatory cytokines (2, 5). Accordingly, in CTL the directional movement of MTOC toward the synapse lasts very few minutes, whereas in long-lived synapses made by Th lymphocytes the MTOC, but also the MVB as we have shown (26), takes from several minutes up to hours to move and dock to the IS (2, 5, 73). Thus, the contribution of the actin reorganization to the polarization of secretory granules is a qualitative feature shared by CTL and Th cells, as well as some of the molecular components that control this reorganization (i.e., WASP, TAGLN2) (3, 71, 74). Nevertheless, there must be quantitative differences in signals, and probably in some molecular mechanisms that govern the actin cytoskeleton dynamics between the cytolytic and helper synapses (5, 75).

Recently, it has been shown in CTL and Jurkat cells that secretory granule convergence toward the MTOC and MTOC polarization to the IS are two mechanistically distinct processes (76). Interestingly, in our analysis of MVB and MTOC migration to the IS at the single cell level, we have found that both MVB and MTOC do not efficiently polarize in PKCδ-interfered cells, but their center of mass converge at nearby positions in control and interfered cells (Figure 1B and Supplementary Figure 1C), correlating in their migration tendency. In addition, our data show that diminished levels of PKCδ did not affect the average centripetal velocity of MVB. Instead, PKCδ appears to specifically regulate the subsequent step of the secretory traffic (i.e., MTOC translocation to the IS). These findings are in part compatible with what was found in PKCδ-KO mouse CTL, in which the lytic granules underwent convergence toward the MTOC, but these granules did not polarize to the IS and the subsequent cytotoxicity was inhibited (29). However, although in these cells the absence of PKCδ inhibited polarization of cytolytic granules, the MTOC polarization toward the IS was not affected (29). Currently, we lack a clear explanation for the observed partial discrepancy, but it is certain that in the mentioned study no simultaneous assessment of MVB and MTOC polarization was performed at the single cell level as we have performed here. Additionally, and most likely, differences between cytotoxic and helper synapses may cause this apparent disagreement (see above).

In conclusion, aside from the well-known role of the PKCθ isotype and the redundant action of PKCε and PKCη isotypes in the polarization of the secretory machinery in CD4^+^ T cells (2, 77), we have identified a positive regulatory role of the PKCδ isotype in MVB polarization and exosome secretion upon IS formation in Th lymphocytes. Regarding the biological significance of such a regulatory mechanism, it is remarkable that melanocytes, among other cell types, undergo multidirectional dispersion of secretory organelles for efficient distribution of their granule contents (78). In these cells, convergence of secretory granules toward the MTOC, which remains at the perinuclear area, prevents (but does not promote) degranulation (78). In contrast, the MTOC polarization to the IS, acting in coordination with the convergence of secretory granules toward the MTOC, is necessary for optimal polarized and focused secretion in many cell types of the immune system, including innate NK cells (79, 80), CTL (4, 76), primary CD4+ T cells (47) and Jurkat cells [(76) and the present report]. This mechanism that now includes the key player PKCδ appears to specifically provide the immune system with a finely-tuned strategy to increase the efficiency of crucial secretory effector functions, while minimizing nonspecific, bystander cytokine stimulation, target cell killing and AICD.

## Author Contributions

VC and MI conceived and designed all the experiments. GH and VC did most of the experiments, analyzed data, and contributed to the writing of the manuscript. PA, SD, BS, DF-M, JG, RdM, MQ, LM-E, AB-G, and TF contributed to the MTOC/MVB polarization experiments and time-lapse studies. AS performed the WB analysis, exosome measurement, and also contributed to rescue experiments. PR-S contributed to actin reorganization experiments, image analysis, statistical analysis of the results, and helped in writing of the manuscript. AF-R contributed to design some experiments and contributed to writing the manuscript. MI conceptualized and coordinated the research, directed the study, analyzed data, and wrote the manuscript. All the authors contributed to the planning and designing of the experiments and to helpful discussions.

### Conflict of Interest Statement

The authors declare that the research was conducted in the absence of any commercial or financial relationships that could be construed as a potential conflict of interest.

## Abbreviations

Ab: antibody
AICD: activation-induced cell death
APC: antigen-presenting cell
C: center of mass
CCH: carbachol
cIS: central region of the immune synapse
Fact-low cIS: F-actin-low region at the center of the immune synapse
CMAC: cell tracker blue-CellTracker™ (7-amino-4-chloromethylcoumarin)
cSMAC: central supramolecular activation cluster
CTL: cytotoxic T lymphocytes
DAG: diacylglycerol
DGKα: diacylglycerol kinase α
dSMAC: distal supramolecular activation cluster
ECL: enhanced chemiluminescence
ESCRT: endosomal sorting complex required for traffic
F-actin: filamentous actin
FasL: Fas ligand
FI: fluorescence intensity
fps: frames *per second*
GFP: green fluorescent protein
HBSS: Hank's balanced salt solution
HRP: horseradish peroxidase
ILV: intraluminal vesicles
IS: immune synapse
MHC: major histocompatibility complex
MIP: maximal intensity projection
MVB: multivesicular bodies
MTOC: microtubule-organizing center
NS: not significant
NTA: nanoparticle tracking analysis
PA: phosphatidic acid
PBL: peripheral blood lymphocytes
PKC: protein kinase C
PKCδ: protein kinase C δ isotype
PKD: protein kinase D
PLC: phospholipase C
PMA: phorbol myristate acetate
Pol. Index: polarization index
pSMAC: peripheral supramolecular activation cluster
PSF: point spread function
ROI: region of interest
SD: standard deviation
shRNA: short hairpin RNA
SEE: Staphylococcus enterotoxin E
SMAC: supramolecular activation cluster
TCR: T-cell receptor for antigen
Th: helper T lymphocyte
TRANS: Transmittance
U.DAG2: Upward DAG2
WB: western blot.
