# Supplementary material for: Protein Kinase C δ Regulates the Depletion of Actin at the Immunological Synapse Required for Polarized Exosome Secretion by T Cells
Source: Front Immunol. 2019 Apr 26;10:851. doi: 10.3389/fimmu.2019.00851 (PMC6499072; doi:10.3389/fimmu.2019.00851)
Supplement: Supplementary file 1 [file Data_Sheet_2.docx]

**Supplementary Material**

**Supplementary Fig. S1. Characterization of control and PKCδ-interfered Jurkat clones and calculation of MTOC and MVB Polarization Indexes. A)** Cell surface levels of CD3/TCR, CD2, CD4, LFA-1, CD28, CD45 and CD95 (Fas) in control and PKCδ-interfered Jurkat stable clones were analyzed by flow cytometry. Table represents the percentage (%) and Mean Fluoescence Intensity (MFI) for each cell surface marker as assessed by flowcytometry. B) The percentage of Jurkat clone cells undergoing conjugate formation after the indicated times of challenge with SEE-pulsed Raji was measured in C3 control and P5 PKCδ-interfered clone cells by time-lapse microscopy, analysing at least 15 microscopy fields per clone and per time point. C) The synaptic conjugates made by the C3 control and the P5 PKCδ-interfered Jurkat clones, with CMAC-labeled, SEE-pulsed Raji cells (blue) were fixed, permeabilized and stained with anti-CD63 (green) and anti-γ-tubulin (magenta) Abs to label MVB and the MTOC, respectively. In the upper scheme, the distances in color (A, cyan; B, green) used for the calculation of Pol. Indexes (A/B) are indicated. The red dot represents the cell center of mass (Cell^c^), whereas the yellow dot indicates the projection of MTOC and MVB center of mass (MTOC^c^ and MVB^c^, respectively, labelled with a cross) on the “B” line. Since the Cell^C^ position was taken as the origin to measure distances, those “A” values in the opposite direction to the synapse were taken as negative. Thus, polarization indexes range form +1 to -1. In the lower panels, this scheme is superimposed on some conjugates (see also Fig. 1B, right panel), to show the calculation of Pol. Indexes in representative synaptic conjugates. The Raji cells and the Jurkat clones are labelled with discontinuous and continuous white lines, respectively. The white arrow indicates the IS area, and the yellow arrow the MTOC. MTOC^c^ and MVB^c^ are labelled with white crosses. D) Calculation of MVB Pol. Index was performed as indicated in panel A for the indicated number of synaptic conjugates made by C3, C7, C9 (control) and S4, P5 and P6 (PKCδ-interfered) clones, that were previously challenged for 1 h with SEE-pulsed Raji cells. Dot plot distribution and average Pol. Index (black horizontal line) are represented. The blue line indicates the cut-off value (Pol. Index = 0.25) used to calculate the percentage of synapses showing polarized MVB. In C3 clone, 39 synapses out of 64 are polarized (polarization efficiency = 61%), whereas in P5 clone only 14 out of 50 synapses are polarized (polarization efficiency = 28%). This figure is related to Fig. 1. Single-factor ANOVA was performed between the indicated groups. NS, not significant; ** p <0.05.

**Supplementary Fig. S2. PKCδ controls MVB polarization in living cells.** A) CMAC-labelled Raji cells (blue) were attached to fibronectin-coated chamber slides and SEE-pulsed (30 min). Synaptic formation by C3 control (upper panel) and P5 PKCδ-interfered (lower panel), CFP-CD63-expressing clones, was imaged by time-lapse microscopy. Representative frames from Suppl. Video 1 are shown (9 min for C3 and 27 min for P5 clone). In the left panels, transmittance and CMAC channels were merged, whereas in the right panels, CMAC and CFP-CD63 channels were merged to visualize the synaptic contact (white arrow) and the MVB (yellow arrow). B) Experiments were performed as in panel A, and the formation of synaptic conjugates made by the C9 control and the S4 PKCδ-interfered Jurkat clones was imaged by long-term time-lapse microscopy. Data is expressed as percentage of synapses with polarized MVB and was calculated as indicated in Materials and Methods and Fig. 1 and Suppl. Fig 1, but analysing the MVB Pol. Index during the entire indicated period. The synapses with MVB substantiating Pol. Indexes >0.25 all along the indicated time were scored as “polarized” and those with Pol. Index <0.25 as “not polarized”. Data are means plus SD (*n* = 3, analyzing at least 19 synapses from 10 different microscopy fields per experiment). Single-factor ANOVA was performed between the indicated groups. ** p≤0.05.

**Supplementary Fig. S3. PKCδ regulates polarized exosome secretion.** C3 control and P5 PKCδ-interfered Jurkat clones were challenged with different stimuli for the indicated culture periods. Subsequently, cells and exosomes were collected and processed as indicated in Material and Methods. A) WB of the cell lysates was performed to analyse PKCδ expression and to normalize exosome secretion by β-actin levels. B) NTA measurements of exosome concentration (particles/ml) were normalized by the exosome-producing cell number, by referring exosome concentration to β-actin signals in the WB of the cell lysates. C) Mean (+SD) size of the exosomes expressed in nm as assessed by NTA analyses of the isolated exosomes. Single-factor ANOVA was performed between the indicated groups. NS, not significant; ** p≤0.05.

**Supplementary Fig. S4. Cell stimulation induces relocalization and enhancement of U.DAG2 fluorescence.** Upper panel, Jurkat J-HM1-2.2 cells expressing either GFP-PKCδ, U.DAG2 or GFP-C1bPKCθ were stimulated with CCH or PMA at the indicated times and imaged by time-lapse fluorescence microscopy. Some representative frames from videos (control, non-stimulated; CCH and PMA) and the corresponding time (min) are represented. Lower panel shows the kinetic analysis of U.DAG2 (continuous line) or GFP-C1bPKCθ (discontinuous line) fluorescence. See also Suppl. Video 4 (first concatenated video) for a representative example. FI ratios were calculated as the mean FI of the cell ROI at different times relative to the mean FI of the same non-stimulated cell (t = 0). Two cells expressing U.DAG2 (continuous lines), and two cells expressing GFP-C1bPKCθ (discontinuous line) are represented. Although the relocalization of GFP-C1bPKCθ to the plasma membrane was observed upon cell activation, the FI ratio corresponding to GFP-C1bPKCθ decreased along time due to photobleaching. In contrast, an enhancement of U.DAG2 FI ratio, associated to its redistribution to the plasma membrane, was observed. The results shown are representative of those obtained with 10 different cells and fields.

**Supplementary Fig. S5. PKD1 and C1bPKC**θ **half-life at the synapse in DGKα-interfered cells.** Jurkat T lymphocytes were co-transfected with DsRed2-PKD1 or GFP-C1bPKCθ together with pSR-GFP or pSR-GFP siRNA-DGKα bicistronic plasmids. A) WB analysis of the cell lysates was performed to analyse DGKα expression. B) Control (DGKα+) and DGKα-interfered (DGKα-) Jurkat cells expressing DsRed2-PKD1 or GFP-C1bPKCθ were challenged with CMAC-labeled, SEE-pulsed Raji cells, and analysed by time-lapse fluorescence microscopy (see Suppl. Video 7). Fluorescence intensity changes over time were analyzed using NIS-AR software on an appropriate ROI at the synaptic region. Half-life of fluorescence decay (mean ± SD) of both DsRed2-PKD1 and GFP-C1bPKCθ at the synapse is shown for DGKα^+^ and DGKα^-^ cells. Single-factor ANOVA was performed between the indicated groups. ** p <0.05.

**Supplementary Fig. S6. IS stimulation induces the increase of U.DAG2 fluorescence intensity.** J-HM1-2.2 cells expressing U.DAG2 were either left unstimulated (control) or challenged with CMAC-labelled (blue), SEE-pulsed Raji cells (synapse). Subsequently, U.DAG2^+^ cells were imaged by time-lapse fluorescence microscopy, and kinetic analysis of U.DAG2 FI ratio was performed as described in Suppl. Fig. 4. Left panel, representative frames at the indicated times (from Suppl. Video 5). Right panel, kinetic analysis of average U.DAG2 FI relative to average U.DAG2 FI at t = 0 for each cell ROI in 2 unstimulated cells (control, discontinuous lines) and 4 cells forming IS (synapse, continuous lines). Figure related to Suppl. Video 5.

**Supplementary Fig. S7. Calculation of the relative area of the F-actin-low region at central synapse.** C3 control and P5 PKCδ-interfered clones were challenged with CMAC-labelled SEE-pulsed Raji cells for 1 h, fixed, immunolabelled with phalloidin and imaged by fluorescence microscopy. A) Top views correspond to the Maximal Intensity Projection (MIP) of the indicated, two merged channels in a representative example. White arrows indicate the direction to visualize the face on views of the synapse (IS interface) enclosed by the boxed ROIs (white rectangles) as shown in Suppl. Video 10. B) The enlarged ROIs (1.5x and 2.5x zoom, respectively) were used to generate the IS interface images shown in the middle panels. The areas of the F-actin-low region at central synapse (Fact-low cIS area) (yellow line) and the synapse (IS area) (white line) were defined and measured as indicated in Material and Methods, and the relative area of the F-actin-low region at cIS (Fact-low cIS area / IS area) was calculated and represented. C) Area ratio dot plot distribution and average area ratio (black horizontal lines) for the indicated number of IS conjugates from one representative experiment are showed. The blue line indicates the cut-off value (area ratio = 0.1) used to calculate the percentage of synapses containing a F-actin-depleted region at cIS, as shown in Fig. 9C. In C3 control clone, 47 synapses out of 59 (80%) show a F-actin-depleted region at the cIS, whereas in P5 PKCδ-interfered clone only 26 out of 54 synapses (48%) show a F-actin-depleted region at cIS. This figure is related to Fig. 9 and Suppl. Video 10. Single-factor ANOVA was performed between the indicated groups. ** p≤0.05.

**Supplementary Video 1.** CMAC-labelled Raji cells (blue) were attached to fibronectin-coated IBIDI chamber slides and pulsed with SEE (30 min). Synaptic formation by C3 control (upper panel) and P5 PKCδ-interfered (lower panel), CFP-CD63-expressing clones, was imaged by time-lapse microscopy. In the left panels, transmittance and CMAC channels were merged, whereas in the right panels, CMAC (blue) and CFP-CD63 (cyan) channels were merged to visualize the synaptic contact (white arrow) and the converged MVB (yellow arrow). The video (2 fps) shows the polarization of MVB towards the synapse contact areas in the C3 control clone, but not in the P5 PKCδ-interfered clone, although both clones formed synaptic conjugates. One representative example is shown out of 27 synapses recorded of each clone. This video is related to Suppl. Fig. 2A.

**Supplementary Video 2.** Upper left panel: C3 control clone co-expressing CFP-CD63 (cyan) and GFP-PKCδ (green) was challenged with CMAC-labelled (blue), SEE-pulsed Raji cells to induce synapse formation. The yellow arrow indicates polarized MVB and the accumulations of GFP-PKCδ. Upper right panel: C3 control clone expressing GFP-PKCδ was challenged with CMAC-labelled (blue), SEE-pulsed Raji cells to induce synapse formation. The yellow arrow indicates the accumulations of GFP-PKCδ. Lower left panel: P6 PKCδ-interfered clone co-expressing CFP-CD63 and very low levels of GFP-PKCδ were challenged with CMAC-labelled (blue), SEE-pulsed Raji cells. The white arrow indicates the synaptic contact. Lower right panel: P6 PKCδ-interfered clone co-expressing CFP-CD63 and GFP-PKCδ forming synapse with CMAC-labelled (blue), SEE-pulsed Raji cells. The white arrow labels the formation of the synaptic contact, whereas the yellow arrow indicates polarized MVB and the accumulations of GFP-PKCδ. In the bottom rows of upper left, and lower panels, transmittance and CMAC channels were merged. The indicated channels were time-lapse imaged (2 fps). Representative examples out of 16 synapses recorded for each condition are shown.

**Supplementary Video 3.** C3 control and P6 PKCδ-interfered Jurkat clones expressing GFP-PKCδ were challenged with CMAC-labelled (blue), SEE-pulsed Raji cells and imaged by time-lapse microscopy (2 fps) to visualize early evidences of AICD upon IS formation. Red arrows indicate the frames at which some features of AICD (plasma membrane blebbing and subsequent cell shrinkage) are observed. Representative examples from at least 35 synapses recorded per clone and transfection are shown.

**Supplementary Video 4.** First concatenated video: J-HM1-2.2 cells expressing U.DAG2 (left panel) or GFP-C1bPKCθ (right panel) were stimulated at the indicated times with Carbachol (CCH, 500 μM) and PMA (100 ng/ml) (2 fps). Second concatenated video: J-HM1-2.2 cells co-expressing CFP-CD63 (left) and U.DAG2 (right) were dropped on anti-TCR coated coverslips and time-lapse microscopy images were acquired (3 fps). **Supplementary Video 5.** J-HM1-2.2 cells co-expressing CFP-CD63 (cyan) and U.DAG2 (green) were challenged with CMAC-labelled (blue), SEE-pulsed Raji cells to induce synapse formation. The synaptic conjugates were imaged by time-lapse microscopy (2 fps) and several frames corresponding to this video are represented in Fig. 5C, left panel. The yellow arrow indicates the accumulations of CFP-CD63 and U.DAG2.

**Supplementary Video 6.** Synapse formed by a Jurkat T lymphocyte co-expressing GFP-PKCδ (green, left) and DsRed2-PKD1 (red, right), challenged with CMAC-labeled, SEE-pulsed (1 μg/ml), Raji cells (blue). The three fluorescence channels were captured (4 fps). In the left panel, merged GFP-PKCδ and CMAC channels, whereas in the right panel merged DsRed2-PKD1 and CMAC channels are shown. DsRed2-PKD1 accumulation was observed at the synaptic contact after 30 min, whereas no accumulation of GFP-PKCδ at the IS was detected.

**Supplementary Video 7.** Jurkat T lymphocytes expressing either DsRed2-PKD1 (red, left) or GFP-C1bPKCθ (green, right) were challenged with a CMAC-loaded, SEE-pulsed (1 μg/ml) Raji cells (blue). The red and green channels were merged to the respective CMAC channel. Transient accumulation at the synaptic membrane of both DsRed2-PKD1 and GFP-C1bPKCθ was imaged (4 fps).

**Supplementary Video 8.** CMAC-labelled Raji cells (blue) were attached to fibronectin-coated IBIDI chamber slides and pulsed with SEE (30 min). Synapse formation by the C3 control clone co-expressing GFP-actin (green) and CFP-CD63 (cyan) was imaged by time-lapse fluorescence microscopy (2 fps). In the left panel, transmittance and CMAC channels were merged, whereas in the central panel, CMAC and GFP-actin channels were merged to visualize the synaptic contact (white arrow) and the cortical actin reorganization at the IS. In the right panel CMAC and CFP-CD63 were merged to visualize the convergence of MVB towards the F-actin-low region at the cIS. The onset of this video (t = 0), showed in thick line in Fig. 8A, upper graphs, correspond to t = 45 min after the addition of C3 clone to the SEE-pulsed Raji cells. Data are representative of the results obtained from at least 19 time-lapse videos.

**Supplementary Video 9.**  CMAC-labelled Raji cells (blue) were attached to fibronectin-coated IBIDI chamber slides and pulsed with SEE (30 min). Synaptic formation by the C3 control (upper panel) and P5 PKCδ-interfered (lower panel), GFP-actin-expressing cells, was imaged by fluorescence time-lapse microscopy (2 fps). In the left panel, transmittance and CMAC channels were merged, whereas in the right panel, CMAC and GFP-actin channels were merged to visualize the synaptic contact (white arrow) and the cortical actin reorganization at the IS. The onset of the videos (t = 0) correspond to t = 45 min (for C3) and t = 30 min (for P5) after the addition of clones to the SEE-pulsed Raji cells showed in Fig. 8A. Data are representative of the results obtained from at least 19 time-lapse videos.

**Supplementary Video 10.** C3 control and P5 PKCδ-interfered clones were conjugated with CMAC-labelled (blue), SEE-pulsed Raji cells. After 1 h of conjugate formation, fixed cells were labelled with phalloidin (green), and analyzed by fluorescence microscopy using the 3D Viewer plugin of the ImageJ software, to visualize Z planes and to project the IS interface. Upper panels: merged CMAC and phalloidin channels. Lower panels: enlarged IS area (1.5x and 2.5x zoom for C3 and P5, respectively, white rectangles in Fig. 9A) of phalloidin channel. Frame no. 1 of the video (2 fps) corresponds to the top view shown in Fig. 9A and, after a 3D rotation, frame no. 44 corresponds to the IS interface view shown in Fig. 9B. The depletion of F-actin at the synaptic central region, is observed in the C3 control clone, but not in the P5 PKCδ-interfered clone. Video is related to Fig. 9.
