# Supplementary material for: Protein Kinase C δ Regulates the Depletion of Actin at the Immunological Synapse Required for Polarized Exosome Secretion by T Cells
Source: Front Immunol. 2019 Apr 26;10:851. doi: 10.3389/fimmu.2019.00851 (PMC6499072; doi:10.3389/fimmu.2019.00851)
Supplement: Supplementary file 12 [file Data_Sheet_1.PDF]

A

| CLON | CD3 | | CD2 | | CD4 | | LFA-1 | | CD28 | | CD45 | | CD95 | |
|---|---|---|---|---|---|---|---|---|---|---|---|---|---|---|
| | % | MFI | % | MFI | % | MFI | % | MFI | % | MFI | % | MFI | % | MFI |
| C3 | 87±2 | 230 | 79±8 | 175 | 69±8 | 125 | 97±2 | 250 | 82±4 | 201 | 98±5 | 650 | 97±5 | 320 |
| C7 | 86±8 | 260 | 81±8 | 160 | 71±8 | 120 | 90±8 | 279 | 86±8 | 190 | 99±8 | 679 | 94±8 | 317 |
| C9 | 94±10 | 210 | 82±7 | 153 | 72±7 | 113 | 94±10 | 240 | 84±8 | 187 | 94±7 | 640 | 93±7 | 305 |
| S4 | 89±6 | 200 | 80±8 | 190 | 70±8 | 120 | 99±6 | 280 | 83±6 | 199 | 99±6 | 680 | 92±6 | 310 |
| P5 | 83±8 | 205 | 85±7 | 145 | 75±7 | 125 | 93±8 | 270 | 81±3 | 186 | 89±8 | 670 | 89±10 | 303 |
| P6 | 87±8 | 240 | 83±6 | 171 | 73±6 | 111 | 97±8 | 257 | 82±4 | 170 | 92±8 | 657 | 95±7 | 295 |
| | NS | NS | NS | NS | NS | NS | NS | NS | NS | NS | NS | NS | NS | NS |

C

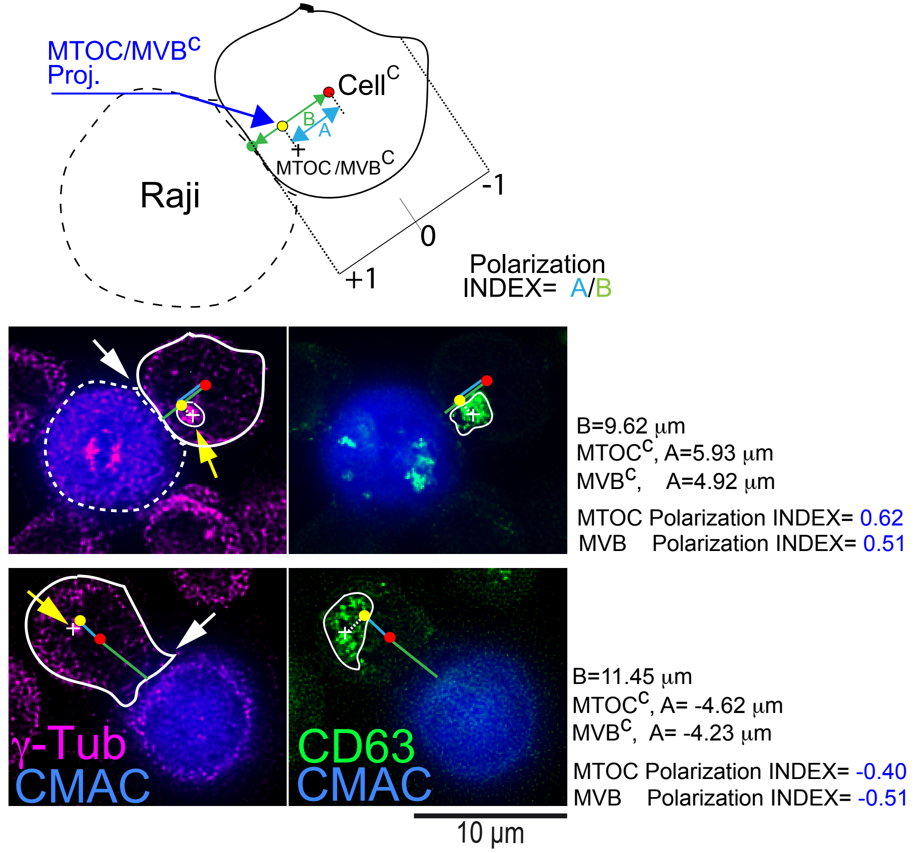


B

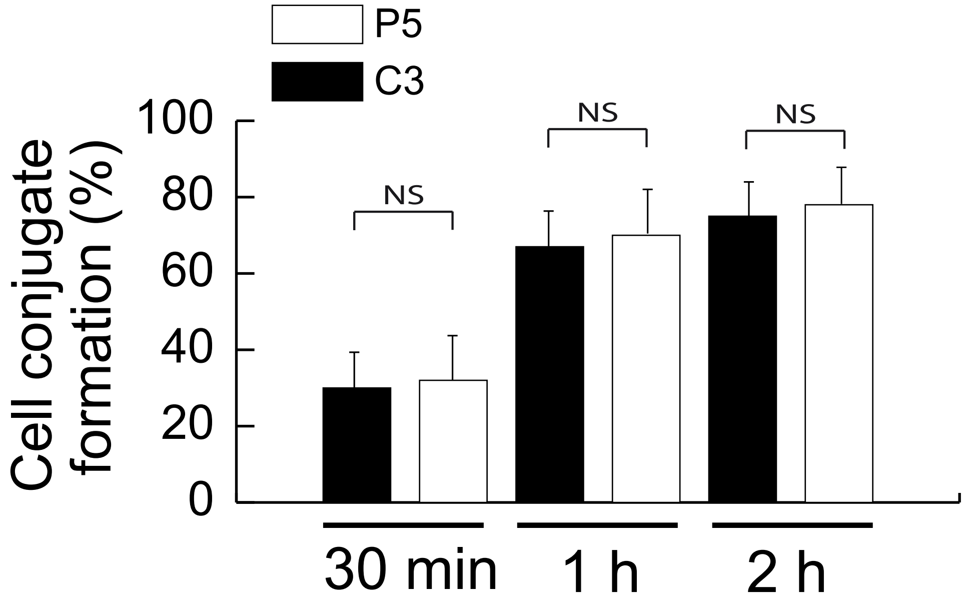


D

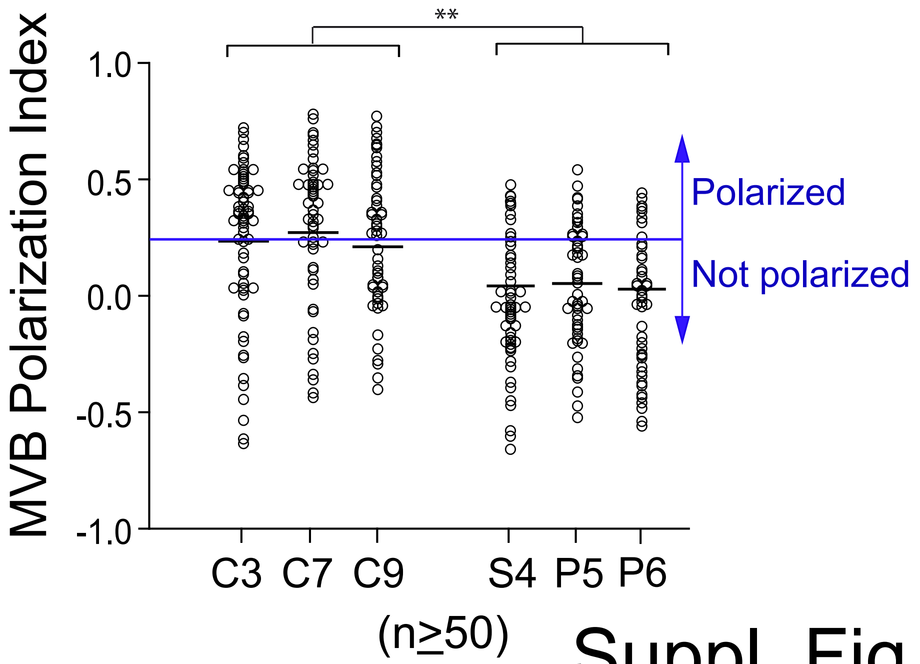


Suppl. Fig. 1

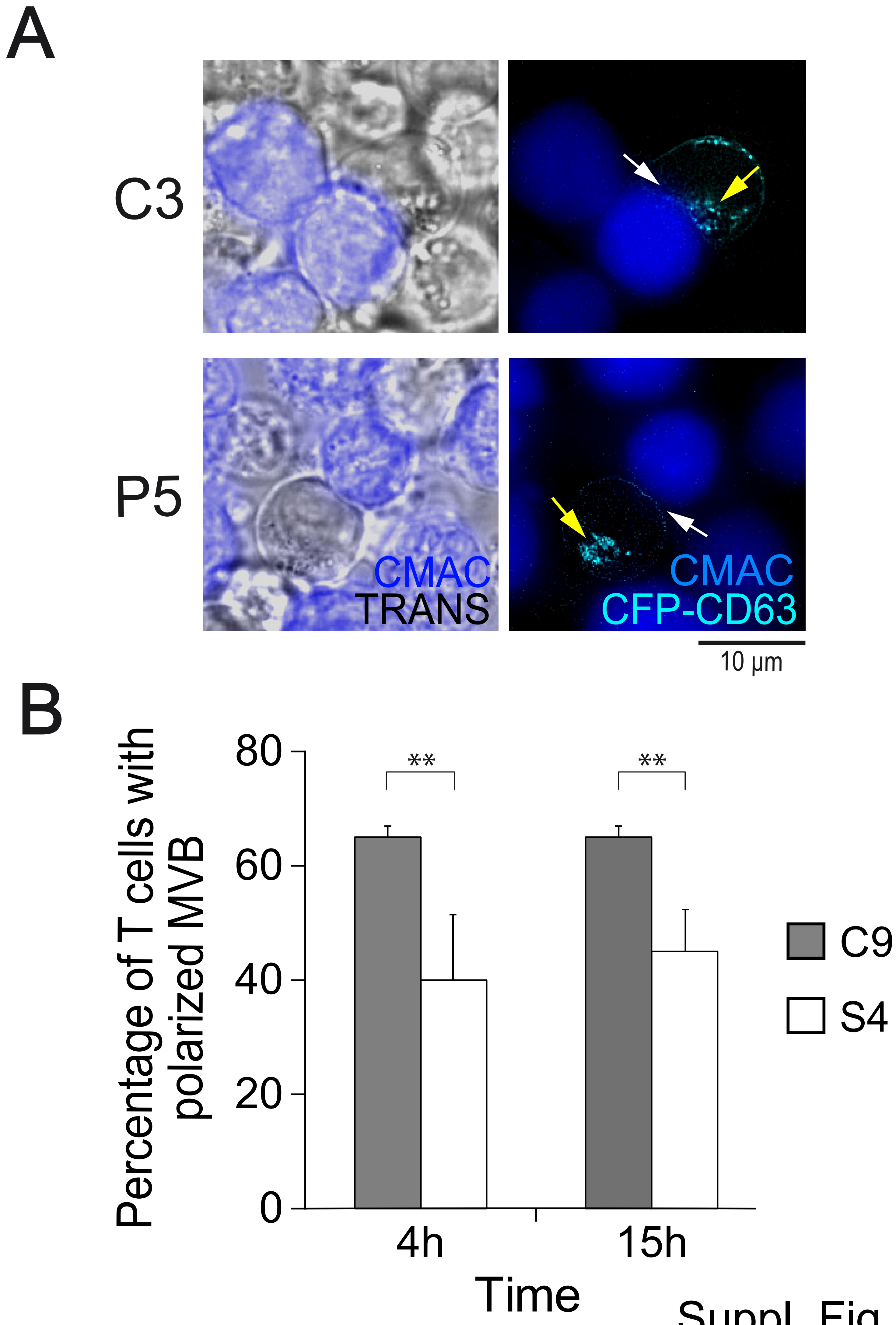

A
C3
P5
CMAC
TRANS
CMAC
CFP-CD63
10 µm
B
Percentage of T cells with polarized MVB
80
60
40
20
0
**
**
4h
15h
Time
C9
S4


Suppl. Fig. 2

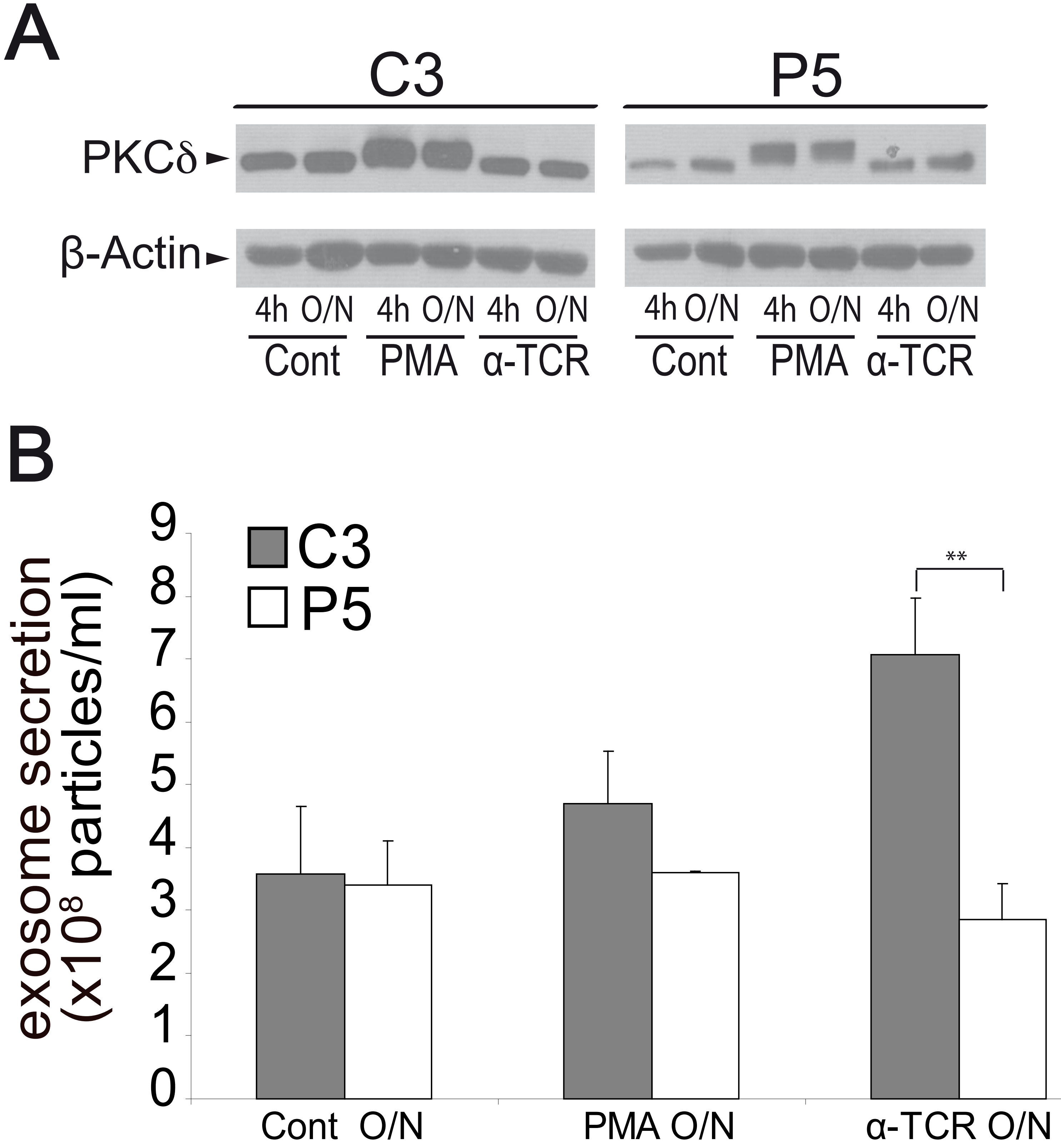


C

| | Cont O/N | PMA O/N | α-TCR O/N |
|---|---|---|---|
| C3 | 225±72 | 227±69 | 209±84 |
| P5 | 232±92 | 239±102 | 233±99 |

NS

Suppl. Fig. 3

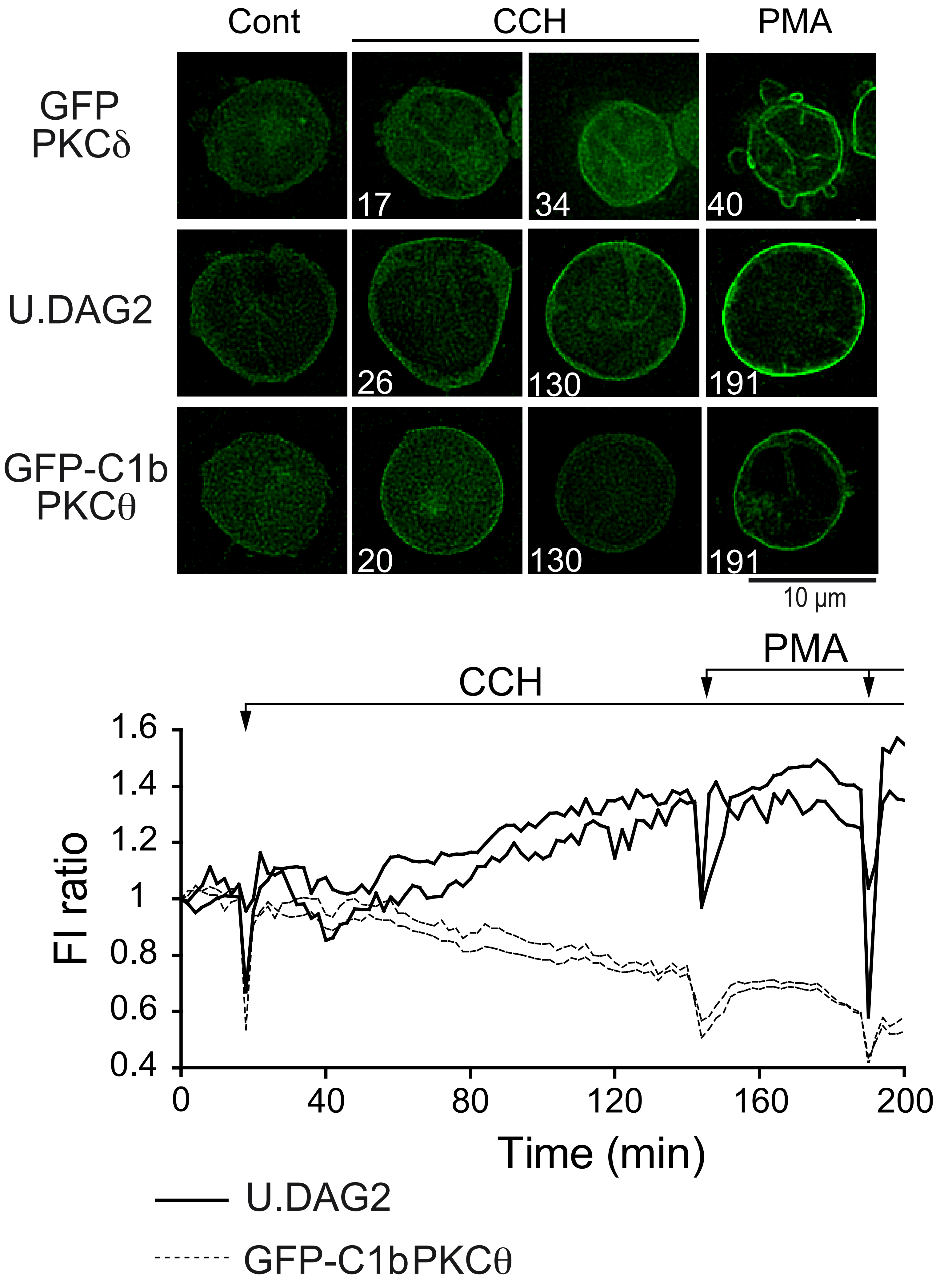

Cont
CCH
PMA
GFP
PKCδ
U.DAG2
GFP-C1b
PKCθ
17
34
40
26
130
191
20
130
191
10 µm
PMA
CCH
FI ratio
1.6
1.4
1.2
1
0.8
0.6
0.4
0
40
80
120
160
200
Time (min)
U.DAG2
GFP-C1bPKCθ


Suppl. Fig. 4

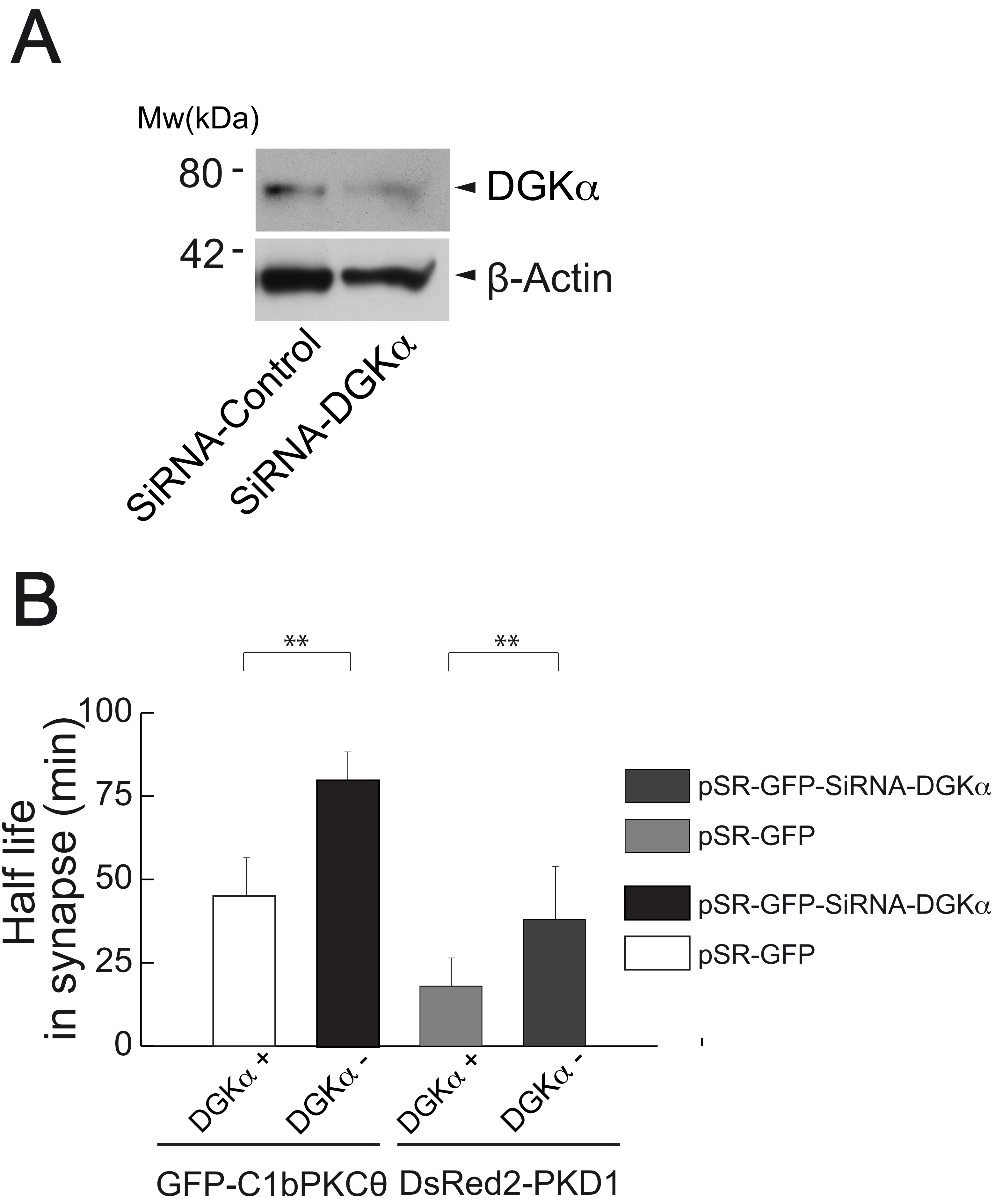
A
Mw(kDa)
80
42
DGKα
β-Actin
SiRNA-Control
SiRNA-DGKα
B
**
**
Half life in synapse (min)
100
75
50
25
0
pSR-GFP-SiRNA-DGKα
pSR-GFP
pSR-GFP-SiRNA-DGKα
pSR-GFP
DGKα +
DGKα -
DGKα +
DGKα -
GFP-C1bPKCθ
DsRed2-PKD1

Suppl. Fig. 5

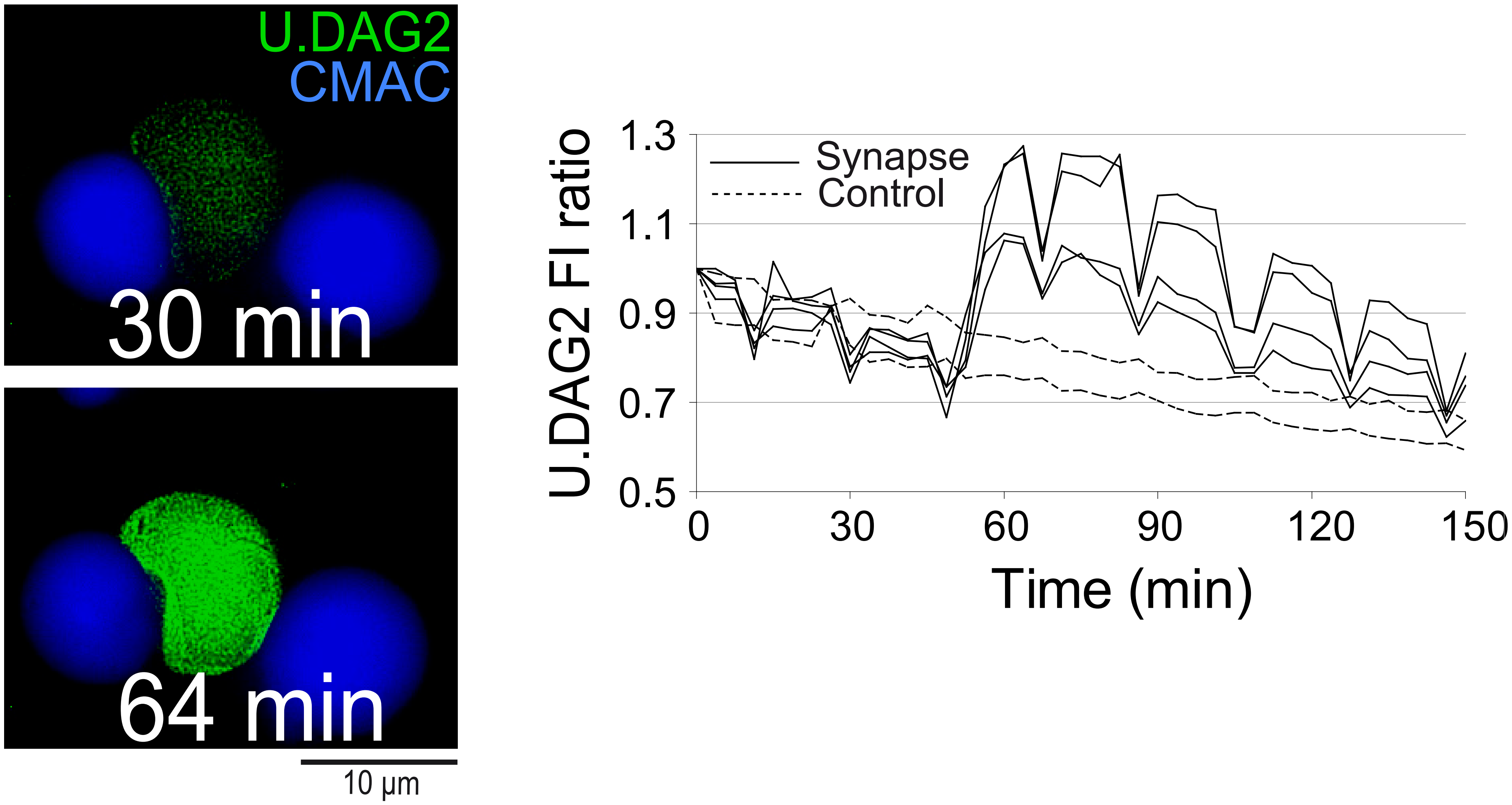
U.DAG2
CMAC
30 min
64 min
10 µm
U.DAG2 FI ratio
Synapse
Control
1.3
1.1
0.9
0.7
0.5
0
30
60
90
120
150
Time (min)

Suppl. Fig. 6

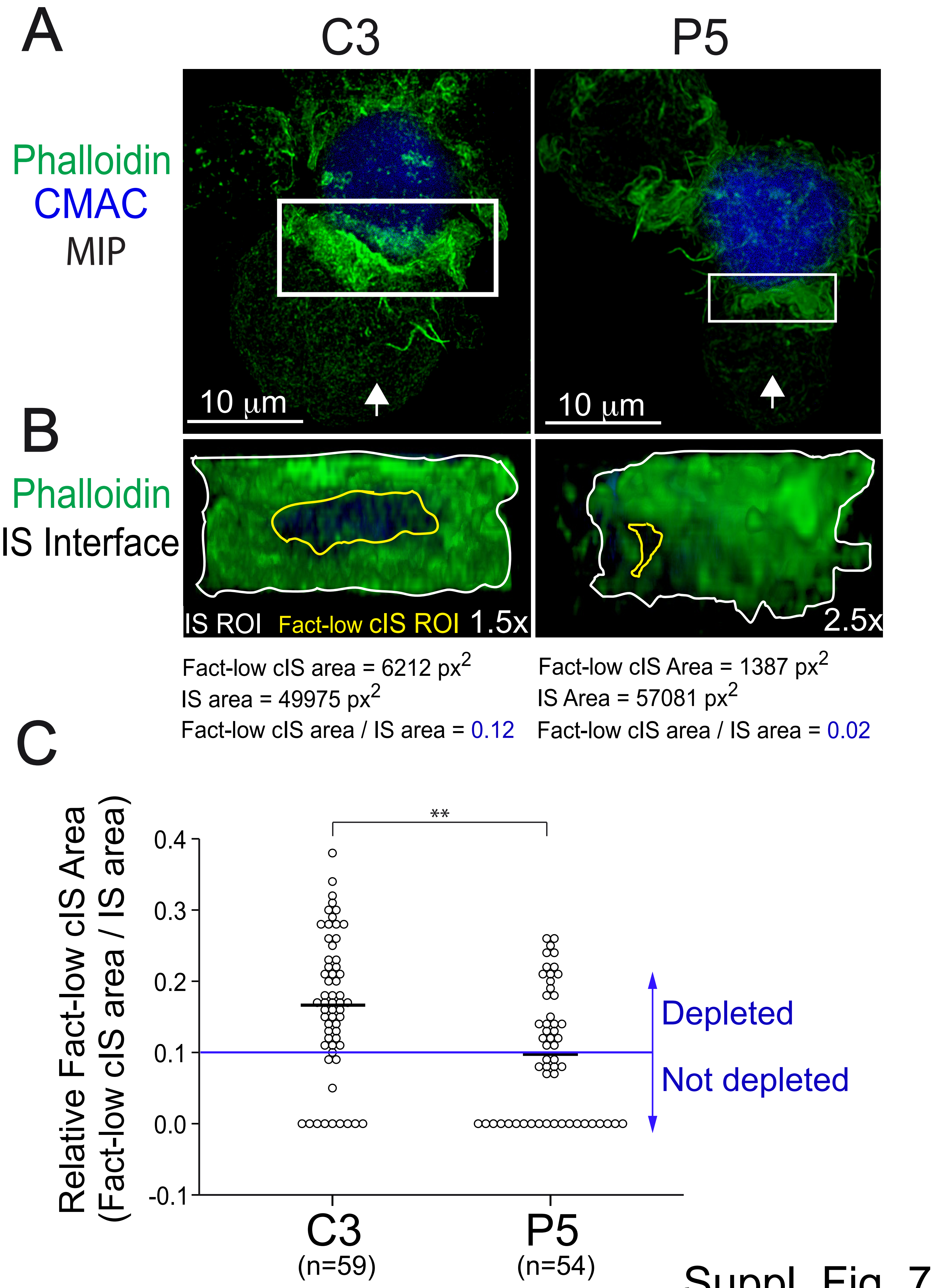
A
C3
P5
Phalloidin
CMAC
MIP
10 μm
10 μm
B
Phalloidin
IS Interface
IS ROI
Fact-low cIS ROI
1.5x
2.5x
Fact-low cIS area = 6212 px$^2$
IS area = 49975 px$^2$
Fact-low cIS area / IS area = 0.12
Fact-low cIS Area = 1387 px$^2$
IS Area = 57081 px$^2$
Fact-low cIS area / IS area = 0.02
C
Relative Fact-low cIS Area
(Fact-low cIS area / IS area)
**
0.4
0.3
0.2
0.1
0.0
-0.1
Depleted
Not depleted
C3
(n=59)
P5
(n=54)

Suppl. Fig. 7
